# A phase I/II study of tagraxofusp in Japanese patients with blastic plasmacytoid dendritic cell neoplasm

**DOI:** 10.1007/s12185-025-04151-5

**Published:** 2026-01-06

**Authors:** Akira Yokota, Wataru Munakata, Toru Kiguchi, Yoshiaki Ogawa, Masayuki Hino, Koji Kato, Masahiro Chiba, Daisuke Kawasaki, Kohei Wasa, Taisuke Mikasa, Kengo Takeuchi, Koji Izutsu, Ritsuro Suzuki, the Study Group, Nobuhiro Hiramoto, Masahiro Onozawa, Senji Kasahara, Noboru Asada, Toko Saito, Toshiro Kawakita, Toshio Kitawaki, Toshihiro Miyamoto, Akihiro Hirakawa, Yasuhito Terui, Junji Suzumiya, Shuichi Miyawaki

**Affiliations:** 1 Department of Hematology, Chiba Aoba Municipal Hospital, 1273-2 Aoba-cho, Chuo-ku, Chiba, Chiba 260-0852 Japan; 2 Department of Hematology, National Cancer Center Hospital, Chuo-ku, Tokyo Japan; 3 Department of Diabetes, Endocrinology and Hematology, Dokkyo Medical University Saitama Medical Center, Koshigaya, Saitama Japan; 4 Department of Hematology and Oncology, Tokai University School of Medicine, Isehara, Kanagawa Japan; 5 Department of Hematology, Graduate School of Medicine, Osaka Metropolitan University, Osaka, Osaka Japan; 6 Department of Medicine and Biosystemic Science, Graduate School of Medical Sciences, Kyushu University, Fukuoka, Fukuoka Japan; 7 Clinical Development Department, Nippon Shinyaku Co., Ltd., Kyoto, Kyoto Japan; 8 Data Science Department, Nippon Shinyaku Co., Ltd., Kyoto, Kyoto Japan; 9 Division of Pathology, the Cancer Institute, Japanese Foundation for Cancer Research, Koto-ku, Tokyo Japan; 10 Pathology Project for Molecular Targets, Cancer Institute, Japanese Foundation for Cancer Research, Koto-ku, Tokyo Japan; 11 Department of Pathology, Cancer Institute Hospital, Japanese Foundation for Cancer Research, Koto-ku, Tokyo Japan; 12 Department of Hematology, Shimane University Hospital, Izumo, Shimane Japan

**Keywords:** SL-401, Blastic plasmacytoid dendritic cell neoplasm, CD123, Tagraxofusp

## Abstract

Tagraxofusp (TAG), a first-in-class CD123-targeted therapy, is a recombinant fusion protein of human interleukin-3 conjugated to a truncated diphtheria toxin payload. Blastic plasmacytoid dendritic cell neoplasm (BPDCN) is an aggressive orphan hematologic cancer with a poor prognosis, and is derived from plasmacytoid dendritic cells that overexpress interleukin-3 receptor subunit alpha (IL3RA or CD123). This open-label phase I/II study evaluated the efficacy and safety of TAG in 11 Japanese BPDCN patients, of whom seven were treatment-naïve (TN) and four had relapsed/refractory (R/R) disease. In the phase I portion, seven patients (five TN, two R/R) were treated, and no dose-limiting toxicity was observed, with 12 μg/kg/day tolerated. In the phase II portion, four patients (two TN, two R/R) were treated. Among the seven TN BPDCN patients, the rate of complete response (CR) + clinical CR (CRc: CR with minimal residual skin abnormality) was 57.1% (90% confidence interval [CI], 22.5–87.1), and the lower limit of the 90% CI (22.5%) exceeded the pre-specified threshold of 10%. Common adverse events included increased alanine aminotransferase (81.8%) and aspartate aminotransferase (72.7%), hypoalbuminemia, hypokalemia, and capillary leak syndrome (54.5%). The results indicate that TAG was effective and had a manageable safety profile in Japanese BPDCN patients.

**Supplementary Information:**

The online version contains supplementary material available at 10.1007/s12185-025-04151-5.

## Introduction

Blastic plasmacytoid dendritic cell neoplasm (BPDCN) is an aggressive orphan hematologic cancer with a poor prognosis that is more common in the elderly and in males [1–4]. BPDCN has a predilection for the skin and may also involve the bone marrow, peripheral blood, lymph nodes, spleen, or central nervous system (CNS). No drug is approved for BPDCN in Japan, and in patients who are eligible for intensive chemotherapy, regimens that are typically used for acute leukemias or lymphomas have been used but with limited therapeutic benefit [5–7].

CD123 is known to be highly expressed on BPDCN cells [8, 9]. Tagraxofusp (TAG), a first-in-class CD123-targeted therapy, is a recombinant fusion protein consisting of human interleukin-3 conjugated to a truncated diphtheria toxin payload [10]. TAG is taken up by cancer cells expressing CD123, the interleukin-3 receptor alpha chain. TAG inhibits protein synthesis in target cells by inactivating elongation factor 2, thereby inducing their apoptosis (programmed cell death) [11].

In a prospective phase I/II study (the 0114 study) of patients with BPDCN, TAG demonstrated a favorable response rate using prespecified multi-system response criteria (57% complete response (CR) + clinical CR (CRc: CR with minimal residual skin abnormality)) in treatment-naïve (TN) patients, 16% CR + CRc in relapsed/refractory (R/R) patients and a favorable safety profile [12, 13]. The duration of response and overall survival (OS) observed with TAG were superior to those reported for existing therapies, which are not approved for the treatment of BPDCN and have not been evaluated in prospective studies using prespecified multi-system criteria [12–19]. Moreover, these therapies are associated with significant toxicity and typically result in relapse within one year [15–19]. Based on the results of the 0114 study, TAG has been approved for adults or pediatric patients aged ≥ 2 years with TN or R/R BPDCN in the US, and for adults with TN BPDCN in Europe. As there is no established standard of care for BPDCN in Japan, there is a clear unmet need for new therapeutic options. To address this, a phase I/II trial was conducted to evaluate the pharmacokinetics (PK), safety, and efficacy of TAG in Japanese patients with BPDCN. This study was registered with the Japan Registry of Clinical Trials (jRCT2031220023).

## Materials and methods

### Eligibility criteria

Eligible patients were aged ≥ 18 years with TN or R/R BPDCN as defined by the 2017 World Health Organization classification with minor modifications [3, 4]. Diagnosis was centrally confirmed by KT, a hematopathologist. Additional inclusion criteria included an Eastern Cooperative Oncology Group (ECOG) performance status (PS) score of 0 to 2. Patients were excluded if they had received chemotherapy within 14 days prior to the start of study treatment, had myocardial impairment resulting in heart failure as determined by New York Heart Association Criteria (Class III or IV), or had CNS involvement of BPDCN. Details of the inclusion and exclusion criteria are provided in the Supplementary Material.

### Study oversight

The study protocol was approved by the Institutional Review Board of each participating hospital, and the study was conducted in accordance with Good Clinical Practice (GCP) guidelines and the Declaration of Helsinki. All patients provided written informed consent before participating in the study.

### Study design and treatment

This open-label, single-arm trial consisted of a phase I portion and a phase II portion. In the phase I portion, PK and safety, including dose limiting toxicity (DLT), were evaluated. In the phase II portion, efficacy and safety were assessed, including data from patients enrolled in the phase I portion. Enrollment into the phase II portion was initiated only after the efficacy and safety evaluation committee confirmed that no safety concerns had arisen based on the adverse events (AEs) that were observed during the first cycle in the phase I portion.

**Fig. 1:**
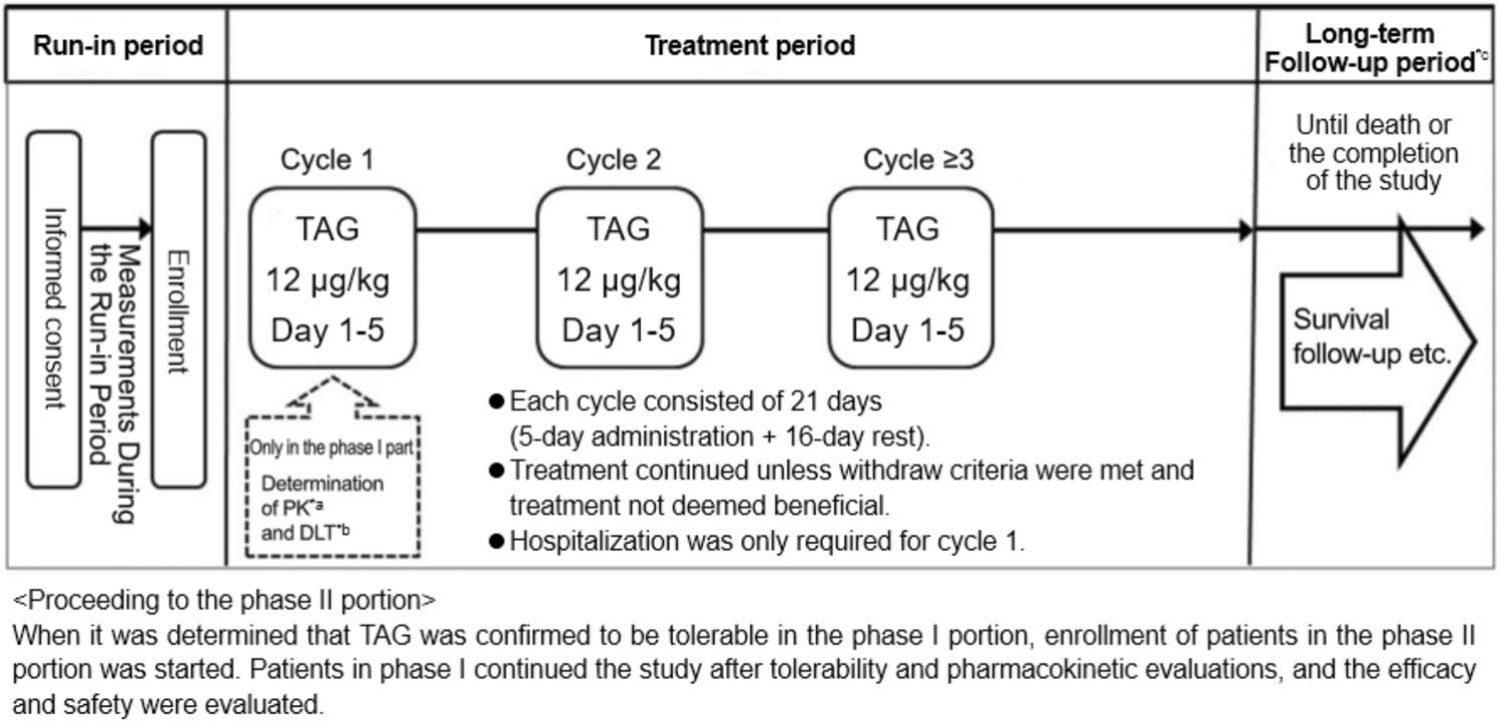
Study design. *TAG* Tagraxofusp; *PK* pharmacokinetics; *DLT* dose-limiting toxicity. *a: Samples were collected on Days 1 and 5 of Cycles 1 and 3. *b: If two of six patients experienced any DLT at 12 μg/kg/day, then the dose was reduced to 9 μg/kg/day. *c: Patients who withdrew during the treatment period were followed up until death or the completion of the study, except in cases where consent was withdrawn or for patients for whom follow up was not possible

The initial dosage of TAG was based on findings from the prior 0114 study, which had demonstrated its efficacy and safety in patients with BPDCN [12, 13]. As no significant differences in the diagnostic criteria, disease severity, treatment practices, or reported racial differences have been identified between Japan and other regions [20], the same dosing regimen was adopted in the current study with appropriate attention to safety. TAG was administered intravenously as a 15-min infusion once daily (on Days 1–5) at a dose of 12 μg/kg, followed by a 16-day treatment-free interval. Each treatment cycle lasted 21 days, and could be repeated. Individual doses of TAG could be delayed but had to be administered within Days 1–10 of the cycle. All patients who discontinued treatment were followed for survival, unless patients withdrew consent or died during the treatment period (Fig. 1).

### End points and assessments

Consistent with the response criteria used in the 0114 study, the International Working Group criteria for AML (2003) [21], the 2007 criteria for assessing response in malignant lymphoma [22], and skin assessments using the modified Severity-Weighted Assessment Tool [23] were utilized as response criteria for determining treatment efficacy for BPDCN in the current study. Detailed criteria are provided in the Supplementary Material. The primary efficacy endpoint was the CR + CRc rate. Secondary efficacy endpoints included median overall survival (OS), duration of CR + CRc, overall response (OR: CR + CRc + complete response with incomplete blood count recovery (CRi) + partial remission (PR)) rate (ORR), duration of response, percentage of patients undergoing hematopoietic stem cell transplantation (HSCT), median progression-free survival (PFS), bone marrow complete response (BMCR) rate, and duration of the BMCR. Safety outcomes included AEs, laboratory tests, vital signs, 12-lead electrocardiograms, body weight, PS and vision tests. AEs were defined as events that occurred from the first administration of TAG until 30 days after the start of the last treatment cycle or treatment discontinuation, regardless of relationship to TAG. The severity of AEs was graded according to the National Cancer Institute Common Terminology Criteria for Adverse Events, version 5.0. DLTs were assessed during Cycle 1 in the phase I portion to determine whether to transition to the phase II portion. The definition of DLTs is provided in the Supplementary Material.

### Pharmacokinetics and immunogenicity

PK samples to determine plasma concentrations and PK parameters of free TAG were collected from seven patients in the phase I portion on Days 1 and 5 of Cycle 1 and Cycle 3: pre-infusion and immediately post-infusion, 15, 30, 45, 60, 90, 120, 180, and 240 min post-infusion. Immunogenicity samples to determine anti-TAG antibodies (ADA), anti-IL-3 antibodies, and anti-TAG neutralizing antibodies (Nab) and anti-IL-3 Nab were obtained from 11 patients on Days 1 (pre-infusion), 15, and 21 of Cycle 1 and on Days 1 (pre-infusion) and 21 of Cycle 2–6. ADA were measured with an electrochemiluminescence assay, anti-IL-3 antibodies were measured with an enzyme-linked immunosorbent assay, and anti-TAG Nab and anti-IL-3 Nab were measured with a cell-based assay. PK parameters were determined using the plasma concentrations of free TAG by non-compartmental PK analysis with Phoenix WinNonlin™ version 8.3 (Certara).

### Statistical analysis

The efficacy analysis set was the full analysis set (FAS) consisting of all treated patients, excluding those who were determined by central review not to meet the diagnostic criteria for BPDCN or who violated GCP guidelines. The primary efficacy analysis was conducted in patients with TN BPDCN. Efficacy was assessed both by site investigators and by an independent review committee (IRC), with the IRC evaluation being considered the primary assessment. The efficacy evaluations used a data cutoff point defined as six months after the initiation of the study treatment in the last enrolled TN BPDCN. In the primary efficacy analysis, the lower bound of the two-sided 90% confidence interval (CI) was calculated by the Clopper–Pearson method. The result was considered statistically significant if the lower bound exceeded the pre-specified threshold of 10%. Safety was evaluated in all treated patients (safety analysis set, SAF).

## Results

### Patient characteristics

**Fig. 2:**
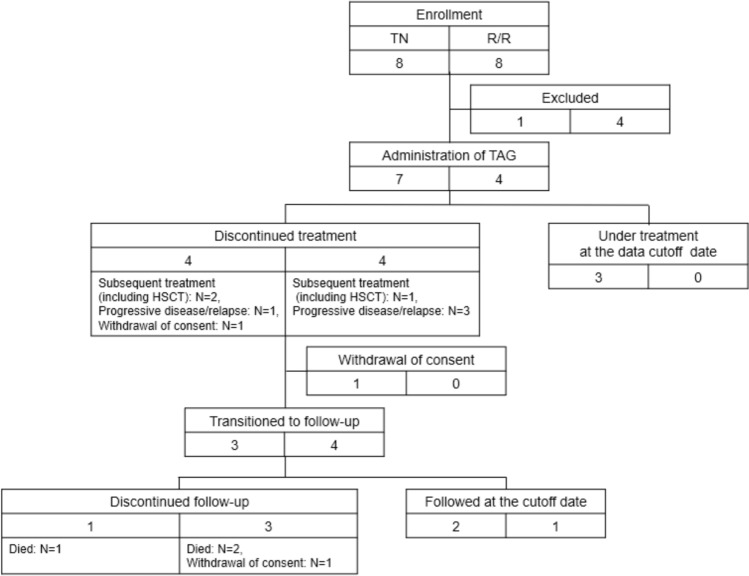
Disposition of patients. *TN* treatment-naïve; *R/R* relapsed/refractory; *HSCT* hematopoietic stem cell transplantation

Patients were enrolled between July 2022 and June 2023 across seven sites in Japan. Eight patients were enrolled in the phase I portion and eight in the phase II portion. Of these, seven patients in the phase I portion and four in the phase II portion received treatment (Fig. 2), resulting in a total of 11 treated patients. All treated patients were included in the FAS. At the data cutoff point, three patients (TN BPDCN) were still receiving treatment, while eight patients (four patients with TN BPDCN and four with R/R BPDCN) had discontinued treatment. In patients with TN BPDCN, the most common reason for discontinuing treatment was initiation of subsequent treatment (two of four [50.0%]) including HSCT in patients with (one of four [25.0%]). For patients with R/R BPDCN, the primary reason for discontinuation was PD or relapse (three of four [75.0%]). As of the data cutoff point, three had died from their primary disease (one with TN BPDCN and two with R/R BPDCN). Baseline characteristics are summarized in Table 1 and Table S1. The median age was 76 years for patients with TN BPDCN and 57 years for patients with R/R BPDCN. The majority of patients were male (six of seven [85.7%] of the patients with TN BPDCN and three of four [75.0%] of the patients with R/R BPDCN). Bone marrow and skin involvement were present in six of the seven (85.7%) patients with TN BPDCN and in all patients with R/R BPDCN. Most patients had involvement of two or more anatomic sites.

**Table 1:** Patient baseline demographic and disease characteristics (FAS)

	Treatment-naïve (N = 7)	Relapsed/refractor (N = 4)
Sex n (%)		
Male	6 (85.7)	3 (75.0)
Female	1 (14.3)	1 (25.0)
Age (year)		
Median	76.0	57.0
Min, Max	58, 86	39, 79
BMI (kg/m^2^)		
Median	26.80	19.95
Min, Max	19.6, 27.3	15.6, 29.1
ECOG PS n (%)		
0	5 (71.4)	3 (75.0)
1	2 (28.6)	1 (25.0)
2	0	0
Affected site at baseline n (%)		
Blood	2 (28.6)	2 (50.0)
Bone marrow	6 (85.7)	4 (100.0)
Head/neck	1 (14.3)	1 (25.0)
Lymph nodes	3 (42.9)	1 (25.0)
Pelvis	0	1 (25.0)
Skin	6 (85.7)	4 (100.0)
Soft tissue	1 (14.3)	0
Spine	0	1 (25.0)
Spleen	1 (14.3)	1 (25.0)
≥ 2 affected sites	6 (85.7)	4 (100.0)
Status of CD123^a^ n (%)		
Positive	7 (100.0)	4 (100.0)
Negative	0	0
Previous lines number of therapy n (%)		
1	NA	2 (50.0)
2	NA	0
3	NA	2 (50.0)

*BMI* body mass index; *ECOG* Eastern Cooperative Oncology Group; *PS* performance status; *NA* not applicable

^a^Assessment of CD123 expression was performed using flow cytometry or immunohistochemistry

### Pharmacokinetics and immunogenicity

**Fig. 3:**
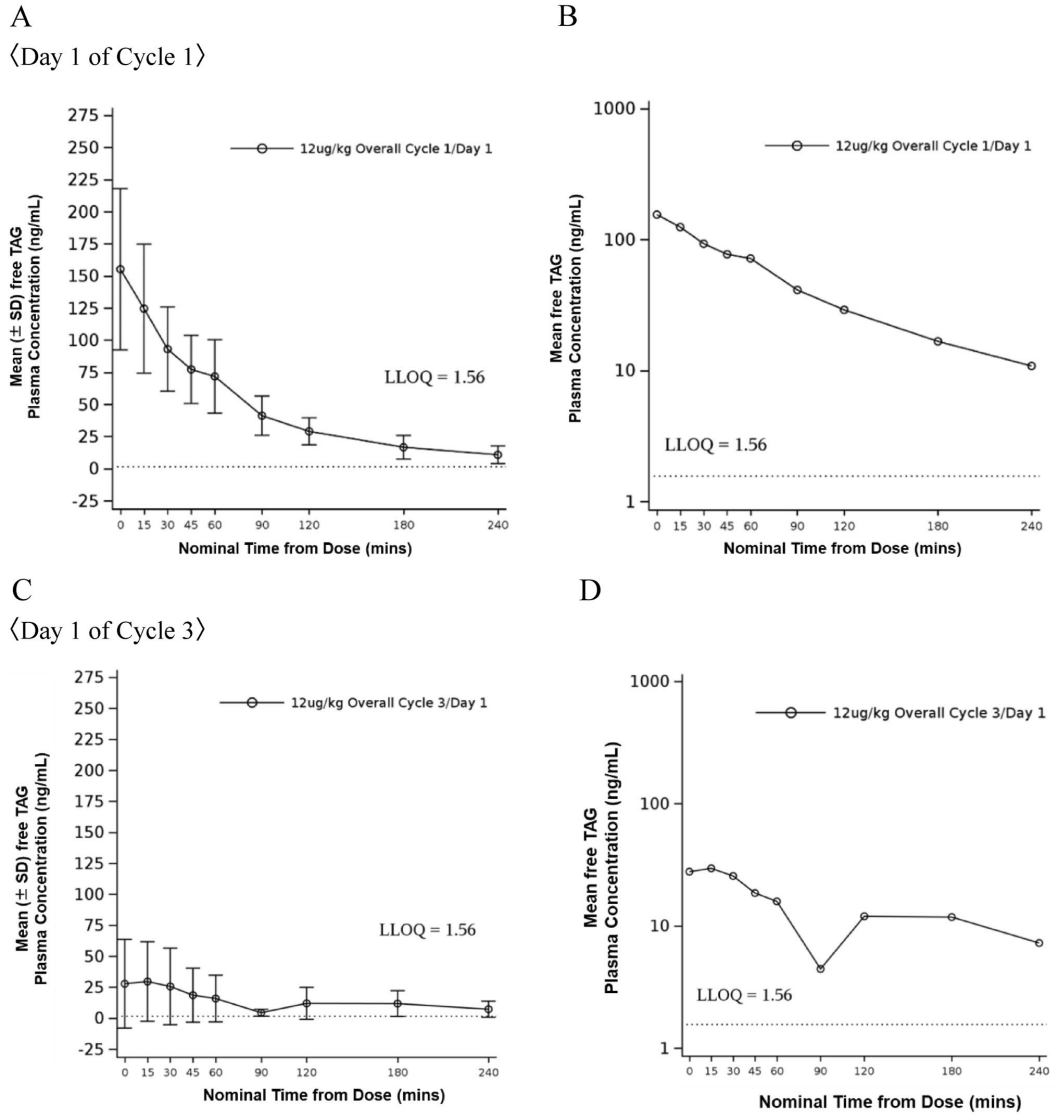
Pharmacokinetics. **A** Plasma concentration changes of free TAG after dosing TAG at 12 μg/kg/day on Day 1 of Cycle 1 (Linear scale). **B** Plasma concentration changes of free TAG after dosing TAG at 12 μg/kg/day on Day 1 of Cycle 1 (Long scale). **C** Plasma concentration changes of free TAG after dosing TAG at 12 μg/kg/day on Day 1 of Cycle 3 (Linear scale). **D** Plasma concentration changes of free TAG after dosing TAG at 12 μg/kg/day on Day 1 of Cycle 3 (Long scale). TAG, Tagraxofusp; LLOQ, lower limit of quantification; LLOQ 1.56 ng/mL. The plasma concentration of free TAG at time 0 indicates the plasma concentration of free TAG immediately after dosing TAG

Plasma concentrations of free TAG following administration of TAG at a dose of 12 μg/kg/day on Day 1 of Cycle 1 and Cycle 3 are shown in Fig. 3. The mean plasma concentration of free TAG on Day 1 of Cycle 1 was higher than that on Day 1 of Cycle 3 at all measured points in time up to 240 min after dosing, indicating reduced exposure to TAG on Day 1 of Cycle 3 compared to Day 1 of Cycle 1 (Fig. 3 A-D). On Day 1 of Cycle 3, TAG concentrations fell below the lower limit of quantification (1.56 ng/mL) at 120 min after dosing in three of the six patients (Fig. 3C). This reduction in TAG exposure was associated with the presence of ADA, with lower TAG concentrations corresponding to higher ADA titers (Table 2). ADA antibody titers increased, and corresponding TAG exposure decreased, in all evaluable patients on Day 1 of Cycle 3 compared to Day 1 of Cycle 1. The rate of increase in ADA titers varied among patients. In 3 patients where ADA was not detected at baseline, the ADA titers on Day 1 of Cycle 3 were 800, 8,000, and 800,000, respectively. In all 11 treated patients, anti-TAG NAb was negative at baseline. Following TAG administration, 90.9% (10 of 11) became positive for anti-TAG NAb (Table 3).

**Table 2:** Mean (SD) of pharmacokinetic parameters by ADA titers at baseline (Day 1 of Cycle 1 and Cycle 3) (SAF)

ADA titers at baseline	Not detected^a^ (N = 3)		8^b^ (N = 1)		80^c^ (N = 1)		800^d^ (N = 2)	
Measurement time point	Cycle 1	Cycle 3	Cycle 1	Cycle 3	Cycle 1	Cycle 3	Cycle 1	Cycle 3
AUC_0-last_^g^ (ng·min/mL)	11,300 (1880)	2690 (4100)	9130 (NA)	1910 (NA)	16,100 (NA)	–	7590 (321)	566 (NA)^e^
AUC_0-inf_^h^ (ng·min/mL)	11,800 (2240)	5190 (6300)^f^	9560 (NA)	2200 (NA)	17,500 (NA)	–	12,200 (6380)	743 (NA)^e^
t_1/2_ (min)	45.5 (17.4)	53.8 (37.0)^f^	54.4 (NA)	83.6 (NA)	62.5 (NA)	–	158 (152)	53.7 (NA)^e^
CL (mL/min/kg)	1.04 (0.218)	8.72 (10.6)^f^	1.26 (NA)	5.46 (NA)	0.688 (NA)	–	1.14 (0.598)	16.1 (NA)^e^
V_z_ (mL/kg)	64.8 (14.9)	394 (354)^f^	98.5 (NA)	658 (NA)	62.0 (NA)	–	195 (114)	1250 (NA)^e^

Mean (SD); *NA* not applicable; *ADA* anti-TAG antibodies; *TAG* Tagraxofusp; Day 1 values for all cycles

^a^The titers on Cycle 3/Day 1 were 800, 8000, and 800,000 in one patient each

^b^The titer on Cycle 3/Day 1 was 8000

^c^Discontinued due to PD (data in Cycle 3 is missing)

^d^The antibody titers in both patients were 8,000,000 on Cycle 3/Day 1

^e^n = 1

^f^n = 2

^g^The observed exposure from the time of TAG administration up to the last quantifiable concentration, relying solely on measured data

^h^The total exposure extrapolated from the time of TAG administration to infinite time. It estimates the overall drug exposure by predicting the elimination profile beyond the last measured time point

**Table 3:** Expression status of ADA, anti-TAG NAb, anti-IL-3 antibodies, and anti-IL-3 Nab before and after TAG administration (SAF)

	ADA (N = 11)		Anti-TAG Nab (N = 11)		Anti-IL-3 antibodies (N = 11)		Anti-IL-3 Nab (N = 11)	
	Pos	Neg	Pos	Neg	Pos	Neg	Pos	Neg
Baseline	8 (72.7)	3 (27.3)	0	11 (100.0)	0	11 (100.0)	0	11 (100.0)
After TAG administration	11 (100.0)	0	10 (90.9)	1 (9.1)	9 (81.8)	2 (18.2)	9 (81.8)	2 (18.2)

n (%); *ADA* anti-TAG antibodies; *Nab* neutralizing antibodies; *Pos* positive; *Neg* negative

### DLTs

Six of the seven patients in the phase I portion were included in the DLT-evaluable population. No DLTs were observed at the recommended Phase 2 dose of 12 μg/kg/day.

### Efficacy

**Fig. 4:**
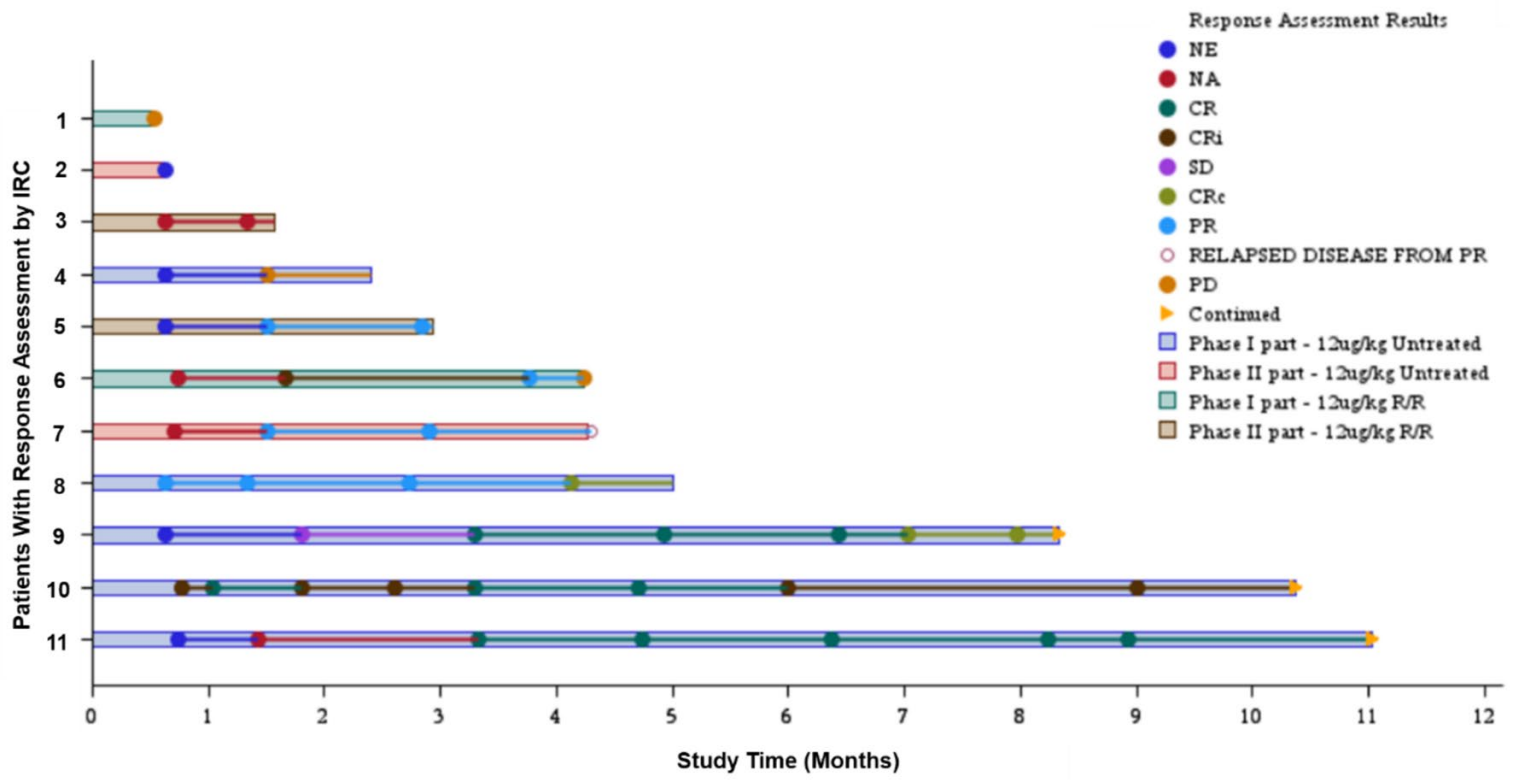
Swimmer’s plot of overall response. *IRC* independent review committee; *NE* not evaluable; *NA* not applicable; *CR* complete remission; *CRi* complete remission with incomplete blood count recovery; *SD* stable disease; *CRc* complete remission with minimal residual skin abnormality; *PR* partial remission; *PD* progressive disease; *R/R* relapsed/refractory. Each horizontal line represents one patient. The length of the bars represents follow-ups through the last assessment by the IRC

In the primary analysis, the CR + CRc rate among the seven patients with TN BPDCN was 57.1% (four of seven), and the lower limit of the 90% CI was at 22.5%, exceeding the pre-specified threshold of 10% (Table 4). IRC assessment of the CR + CRc rate was consistent with that of the site investigator’s assessment. The median follow-up for the primary outcome was 8.5 months (range, 0.6–12.5 months), and the median duration of CR + CRc could not be estimated at the data cutoff point. However, all responding patients maintained their response as of that point in time (Fig. 4). Median OS was also not reached (Table S2), but one patient died at six months due to their primary disease, and no early mortality signals were observed. ORR and BMCR rates were 71.4% (five of seven) and 66.7% (four of six), respectively (Table 4, Table S2). Median durations for response, BMCR, and PFS could not be estimated due to the limited number of events at the data cutoff point. Among the seven patients with TN BPDCN, only one underwent HSCT (Table S2), likely due to the advanced age of the patient. Nevertheless, at the data cutoff point, response was ongoing in all three patients who achieved CR and did not undergo HSCT.

**Table 4:** Remission rate (IRC assessment, FAS)

	Treatment-naïve (N = 7)	Relapsed/refractory (N = 4)
CR + CRc rate		
n (%)	4 (57.1)	0
90%CI	22.53, 87.12	NA
Overall response (CR + CRc + CRi + PR) rate		
n (%)	5 (71.4)	2 (50.0)
90%CI	34.13, 94.66	9.76, 90.24
Best overall response n (%)		
CR	3 (42.9)	0
CRc	1 (14.3)	0
CRi	0	1 (25.0)
PR	1 (14.3)	1 (25.0)
SD	0	0
PD	1 (14.3)	1 (25.0)
Not evaluable	1 (14.3)^a^	0
Not applicable	0	1 (25.0)^b^

*CR* complete remission; *CRc* complete remission with minimal residual skin abnormality; *CRi* complete remission with incomplete blood count recovery; *PR* partial remission; *SD* stable disease; *PD* progressive disease

^a^Because of early discontinuation due to consent withdrawal, no efficacy evaluation of skin and lymph node lesions was performed

^b^CR, CRi, CRc, or PR were not achieved with no progress observed, but the duration was less than eight weeks

No patients with R/R BPDCN achieved CR or CRc. However, CRi was observed in one patient, and another achieved a PR, indicating some level of efficacy (Table 4).

### Safety

A total of 11 patients were included in the SAF (seven TN and four R/R). All patients had at least one AE, and seven patients (63.6%) had serious AEs. Common AEs are summarized in Table 5. The most frequently reported non-hematologic AEs (frequency of ≥ 50% of patients) were elevated alanine aminotransferase (ALT) (81.8%), elevated aspartate aminotransferase (AST) (72.7%), hypoalbuminemia (54.5%), hypokalemia (54.5%), and capillary leak syndrome (CLS: 54.5%). The most frequently reported Grade ≥ 3 AEs were elevated AST (63.6%) and elevated ALT (54.5%), and the most frequent serious AE was capillary leak syndrome (36.4%). However, all of these events were reversible and manageable with dose interruptions or symptomatic treatments. Most cases of CLS were Grade 2 in severity (81.2%), and no patients died due to CLS (Table S3). Most CLS events were observed during or within a few days after Cycle 1 and 2 administrations, and symptoms generally resolved within approximately two weeks following treatment interruption or appropriate supportive care.

**Table 5:** Adverse event incidence (SAF)

	All patients (N = 11)	Treatment-naïve (N = 7)	Relapsed/refractory (N = 4)
All adverse events	11 (100.0)	7 (100.0)	4 (100.0)
Hematologic adverse events^a^			
Anaemia	5 (45.5)	3 (42.9)	2 (50.0)
Disseminated intravascular coagulation	2 (18.2)	1 (14.3)	1 (25.0)
Neutropenia	2 (18.2)	2 (28.6)	0
Thrombocytopenia	2 (18.2)	1 (14.3)	1 (25.0)
Non-hematologic adverse events^a^			
Alanine aminotransferase increased	9 (81.8)	5 (71.4)	4 (100.0)
Aspartate aminotransferase increased	8 (72.7)	4 (57.1)	4 (100.0)
Hypoalbuminemia	6 (54.5)	6 (85.7)	0
Hypokalemia	6 (54.5)	5 (71.4)	1 (25.0)
Capillary leak syndrome	6 (54.5)	4 (57.1)	2 (50.0)
Nausea	5 (45.5)	3 (42.9)	2 (50.0)
Pyrexia	5 (45.5)	3 (42.9)	2 (50.0)
Neutrophil count decreased	5 (45.5)	3 (42.9)	2 (50.0)
Platelet count decreased	5 (45.5)	3 (42.9)	2 (50.0)
Hyperuricemia	4 (36.4)	3 (42.9)	1 (25.0)
Blood lactate dehydrogenase increased	3 (27.3)	1 (14.3)	2 (50.0)
Atrial fibrillation	2 (18.2)	1 (14.3)	1 (25.0)
Constipation	2 (18.2)	2 (28.6)	0
Vomiting	2 (18.2)	1 (14.3)	1 (25.0)
Malaise	2 (18.2)	1 (14.3)	1 (25.0)
Blood creatinine increased	2 (18.2)	2 (28.6)	0
Hyponatremia	2 (18.2)	1 (14.3)	1 (25.0)
Tumour lysis syndrome	2 (18.2)	2 (28.6)	0
Rash	2 (18.2)	2 (28.6)	0
Grade ≥ 3 adverse event^b^	11 (100.0)	7 (100.0)	4 (100.0)
Alanine aminotransferase increased	7 (63.6)	3 (42.9)	4 (100.0)
Aspartate aminotransferase increased	6 (54.5)	3 (42.9)	3 (75.0)
Neutrophil count decreased	5 (45.5)	3 (42.9)	2 (50.0)
Platelet count decreased	4 (36.4)	2 (28.6)	2 (50.0)
Anaemia	3 (27.3)	1 (14.3)	2 (50.0)
Hypokalemia	3 (27.3)	3 (27.3)	0

n (%); adverse events were coded using MedDRA ver. 26.1

^a^Adverse events of any grade with an incidence of at least 18.2% (two out of 11 patients) are listed

^b^Adverse events of Grade 3 or higher, with an incidence of at least 27.3% (three out of 11 patients) are listed

There were no AEs leading to death, treatment discontinuation, or dose reduction. AEs leading to dose interruption were reported in 90.9% (10 of 11). The most frequently reported AEs leading to a dose interruption were elevated AST (54.5%), hypoalbuminemia (54.5%), CLS (54.5%) and elevated ALT (45.5%). However, the administration of TAG was resumed after a dose interruption except in one patient (R/R BPDCN) who discontinued the study due to PD. No AEs directly led to treatment discontinuation. Most AEs occurred during the first two treatment cycles and tended to decrease in frequency with subsequent cycles.

## Discussion

The favorable efficacy and manageable safety profile of TAG in patients with BPDCN was established in the 0114 study [12, 13]. The present phase I/II study was conducted to evaluate whether TAG is similarly effective and safe in Japanese patients with BPDCN.

In this study, most patients were elderly and male, with the primary affected sites being skin and bone marrow, reflecting a typical clinical presentation of BPDCN. No notable differences in baseline characteristics were observed between this study and the 0114 study [12, 13].

Of the 11 patients in this study, eight were ADA-positive at baseline, while the remaining three initially ADA-negative patients developed ADA after administration of TAG. The high prevalence of ADA-positive at baseline was likely due to standard administration of the diphtheria vaccine in Japan. Although exposure to TAG decreased over time due to rising ADA titers, no clinically meaningful impact on efficacy or safety was observed. On Day 1 of Cycle 3, three patients (#4, #8, and #10 in Fig. 4) exhibited TAG concentrations below the lower limit of quantification (1.56 ng/mL) at 120 min post-dose. Notably, patient #8 achieved CRc by Cycle 6 and subsequently underwent HSCT. Furthermore, patient #10 achieved CR by Cycle 4 and maintained remission at the data cutoff point. TAG has a short half-life and likely exerts its effects rapidly by binding to receptors and inducing internalization. This rapid cellular engagement, combined with its femtomolar potency against BPDCN cells and the mechanism of action where a single molecule is hypothesized to be sufficient for cell death, enables TAG to exert potent efficacy even with minimal and transient exposure [24, 25]. Therefore, the low PK may not indicate reduced exposure, but rather suggest that the exposure is appropriate for efficacy and tolerable safety. Additionally, hypersensitivity adverse events were observed in 45.5% (5 of 11) patients with BPDCN, though no events of Grade 3 or higher were reported (Table S4). No association was found between elevated ADA antibody titers and an increased risk or severity of hypersensitivity adverse events.

The CR + CRc rate of patients with TN BPDCN in the current study was 57.1% (four of seven), comparable to the rate in the 0114 study (56.9%; 37 of 65), indicating a similarly high response rate in Japanese patients with BPDCN [13]. The median duration of CR + CRc in this study could not be estimated at the data cutoff point; in contrast, the median duration of CR + CRc was 24.9 (95% CI, 3.8-not reached) months in the 0114 study, with a 24-month probability of sustained CR + CRc of 53% [13]. Although complete responses have been reported with other induction therapies, albeit not in a prospective manner with pre-specified multisystem response criteria, early relapse is common, with a median OS of 7–13 months [14–19]. In the current study, median OS was not reached at the data cutoff point. In the 0114 study, the median OS was 15.8 (95% CI, 9.7–25.8) months; in addition, the estimated survival rate at 24 months was 40% [13], indicating favorable long-term outcomes with TAG.

In patients with BPDCN, who have had historically poor outcomes [26, 27], HSCT is recommended following the achievement of CR [18, 28, 29], with curative potential. In the 0114 study, overall, a total of 32% (21 of 65) of TN BPDCN patients and a total of 51% of patients who achieved CR/CRc were bridged to HSCT directly following treatment with TAG and TAG-induced response. Among patients who underwent HSCT, the median OS was 38.4 months (range, 3.4–58.1), and 72% remained in remission for 12 months post-HSCT [13]. In the current study, the proportion of patients undergoing HSCT was lower than what was seen in the 0114 study, likely due to advanced age; all responding patients were aged 70 years or older, except for one patient (age 61) who underwent HSCT. This contrasts with the 0114 study, where the median age of patients who underwent HSCT was 63 (range, 22–75). The single patient with TN BPDCN who underwent transplantation after achieving CRc in the current study maintained remission and was alive at the data cutoff point. Despite not undergoing HSCT in the current study, three patients with TN BPDCN who achieved CR remained on treatment and maintained their responses for over five months at the data cutoff point. Notably, in the 0114 study, four of the 18 patients who achieved CR + CRc but did not undergo HSCT maintained a response longer than six months, including one patient with a 27 month response and another with a 52 month response [13]. These findings suggest that durable responses can be achieved even in patients who are not candidates for HSCT. Taken together, these results indicate that TAG may offer the potential for long-term disease control in Japanese patients with BPDCN. However, given the limited follow-up duration in the current study, longer follow-up and further analyses are needed to fully assess the long-term efficacy of TAG in Japanese patients.

There were only four patients with R/R BPDCN in the current study, and that number is not sufficient to evaluate the efficacy of treatment in this population. CR or CRc was not achieved in any patient with R/R BPDCN in the current study, but a certain degree of efficacy was shown as one patient achieved CRi and one patient achieved PR. Furthermore, the 0114 study reported an ORR of 58% (95% CI, 33.5–79.7) in the 19 patients with R/R BPDCN, with one patient achieving CR and two patients achieving CRc. Considering that R/R BPDCN has a very poor prognosis compared to TN BPDCN and that no effective treatment has been established at this time, the results of the current study are clinically relevant, and TAG may be a new treatment option for BPDCN in Japan.

The safety profile in this study was consistent with that of the 0114 study [12, 13], and no Japanese-specific safety signals were identified. One AE that warrants particular attention when administering TAG is CLS. TAG may induce CLS through multiple pathways, including direct damage to vascular endothelial cells by its diphtheria toxin payload, cytokine storm triggered by rapid tumor cell lysis [30, 31]. These mechanisms primarily increase vascular endothelial cell permeability, leading to vascular leakage. While the 0114 study resulted in three deaths related to CLS [13], there have been no deaths due to CLS reported in two recent real-world studies in Europe, where CLS management guidelines have been implemented [32, 33]. Albumin levels decrease in most patients soon after TAG administration, requiring close monitoring of albumin levels. In the current study, CLS was most frequently observed during or within a few days after Cycle 1 and 2, and generally resolved within about two weeks with drug interruption or appropriate supportive care (Figure S1−5). In the 0114 study, CLS occurred predominantly in Cycle 1, with all but one event occurring within that cycle, and the median time to CLS onset from therapy initiation was six (range, 3–51) days [13]. Albumin monitoring and supplementation are critical to CLS identification and management. For patients with decreased albumin levels in the current study, a prophylactic measure for CLS was implemented: albumin was administered until the serum albumin level reached ≥ 3.5 g/dL and < 0.5 g/dL below that at the start of the cycle. Additional research exploring identification of potential biomarkers for CLS prediction and monitoring may aid in establishing an optimal approach to CLS prevention. Early detection is a critical feature of CLS management. In both the US and EU, the CLS management guide for TAG developed by Stemline Therapeutics is used to manage CLS, including albumin levels. In addition, hepatic dysfunction was frequently observed as a major AE that was Grade 3 or higher, so AST and ALT levels should be carefully monitored (Figure S6-7). However, most AEs were non-serious, reversible, and manageable with a dose interruption. Most AEs occurred during the early cycles of TAG administration, with no evidence of cumulative toxicity. CNS involvement was observed in one patient (R/R BPDCN) who discontinued the study due to PD. The risk of CNS involvement has been reported for BPDCN [34], and international guidelines recommend intrathecal (IC) doses of methotrexate, cytarabine, and steroids prophylactically to prevent CNS involvement or to treat active CNS, in combination with BPDCN treatment. This was successful in real-world practice where concomitant use TAG and IC led to complete responses in patients with BPDCN, CNS clearance and subsequent HSCT [32, 33].

A limitation of this study was the small number of patients. To validate these results, further analysis of efficacy and safety using larger real-world data is warranted. The diagnosis of BPDCN may vary among physicians, so patients should be carefully evaluated to receive this drug.

In conclusion, this phase I/II study suggested that TAG is effective in Japanese patients with both TN and R/R BPDCN as well as with a manageable safety profile. The results of this study suggest a clinical benefit with TAG in Japanese patients with BPDCN.

## Supplementary Information

Below is the link to the electronic supplementary material.

Supplementary file1 (DOCX 30846 KB)

## Declarations

### Data availability

All data generated or analyzed during this study are included in this published article and its supplementary information files.
