# Supplementary material for: A phase I/II study of tagraxofusp in Japanese patients with blastic plasmacytoid dendritic cell neoplasm
Source: Int J Hematol. 2026 Jan 6;123(5):708–19. doi: 10.1007/s12185-025-04151-5 (PMC13171773; doi:10.1007/s12185-025-04151-5)
Supplement: Supplementary file 1 — Supplementary file1 (DOCX 30846 KB) [file 12185_2025_4151_MOESM1_ESM.docx]

**Supplementary Material**

**Inclusion and exclusion criteria**

[Inclusion criteria]

Patients who met all of the following requirements were considered for admission to the study:

1. The patient has been histologically or cytologically diagnosed with BPDCN according to the 2017 WHO Classification and has either

- Previously untreated BPDCN, or

- Evidence of persistent or recurrent BPDCN in the peripheral blood, bone marrow, spleen, lymph nodes, and/or skin following at least one regimen of systemic therapy (e.g., HSCT and chemotherapy [excluding hydroxyurea]).

1. The patient is a Japanese male or female who is ≥18 years old when giving informed consent.
2. The patient has an ECOG PS score of 0-2.
3. The patient has adequate baseline organ function, including cardiac, renal, and hepatic function, as demonstrated by the latest laboratory values observed within 14 days (28 days for LVEF) before the start of study treatment that are within the following ranges:

- LVEF ≥ the LLN as measured by echocardiography or MUGA scan and no clinically significant abnormalities that require treatment on a standard 12-lead ECG

- Serum creatinine ≤1.5 mg/dL

- Albumin ≥3.2 g/dL (Albumin supplementation to meet this albumin criterion is not permitted.)

- Total bilirubin ≤1.5 mg/dL

- AST and ALT ≤2.5 times the ULN

1. The patient has signed an informed consent form for participation in this study.
2. The patient is able to adhere to this clinical study protocol.
3. The patient agrees to use acceptable contraceptive methods (tools and/or medications) from the start until 2 months after completion/termination of the treatment period (this requirement is not applied to a female of no childbearing potential because of menopause [at least one year has elapsed after her last menstrual period without any other medical reason], because of having undergone hysterectomy or bilateral oophorectomy, or for any other reason.)
4. The patient has a life expectancy of at least 12 weeks after receiving the first tagraxofusp infusion in the opinion of the investigator/sub-investigator.

[Exclusion criteria]

Patients who have any of the following conditions were excluded from this study:

- 1. The patient has persistent clinically significant CTCAE Grade ≥2 toxicities from previous chemotherapy (excluding alopecia, nausea, fatigue, and hepatic function abnormal).
  2. The patient has received treatment with chemotherapy, wide-field radiation, or biologic therapy within 14 days of the start of study treatment.
  3. The patient has undergone HSCT within one year of the start of study treatment.
  4. The patient has received treatment with another investigational product or any unapproved drug in Japan within 14 days of the start of study treatment.
  5. The patient has an active malignancy and/or cancer history within two years of the start of study treatment that may confound the assessment of the study endpoints. (Patients with the following neoplastic diagnoses are eligible: non-melanoma skin cancer, carcinoma in situ, cervical intraepithelial neoplasia, and organ-confined prostate cancer with no evidence of progressive disease.)
  6. The patient has a clinically significant cardiovascular disease (e.g., uncontrolled or any NYHA Class III or IV congestive heart failure, uncontrolled angina, history of myocardial infarction within 180 days of the start of study treatment, history of unstable angina or stroke, uncontrolled hypertension or clinically significant arrhythmia not controlled by medication).
  7. The patient has an uncontrolled, clinically significant respiratory disease (e.g., chronic obstructive pulmonary disease and pulmonary hypertension) that, in the opinion of the investigator (subinvestigator), would put the patient at significant risk for pulmonary complications during the study.
  8. The patient has CNS involvement of BPDCN.

If suspected, CNS involvement of BPDCN should be ruled out with relevant imaging and/or examination of cerebrospinal fluid.

- 1. The patient is receiving immunosuppressive therapy, with the exception of low-dose (≤10 mg/day) prednisolone for treatment or prophylaxis of GVHD.

If the patient has been on immunosuppressive treatment or prophylaxis for GVHD, the treatment(s) must have been discontinued at least 14 days prior to the start of study treatment, and there must be no evidence of CTCAE Grade ≥2 GVHD.

- 1. The patient has uncontrolled intercurrent illness (including uncontrolled infection, disseminated intravascular coagulation [DIC], or psychiatric illness that would limit compliance with protocol requirements).
  2. The patient has known positive status for human immunodeficiency virus (HIV), or active or chronic hepatitis B or hepatitis C.
  3. The patient has continuously been oxygen-dependent.
  4. The patient has a history of hypersensitivity reactions to any ingredients of tagraxofusp or proteins derived from *Escherichia coli*.
  5. The patient has a confirmed or potential pregnancy or is breastfeeding.

A female patient should undergo pregnancy testing during the run-in period. This will not be required for patients of non-childbearing potential such as those who are postmenopausal (at least one year has elapsed after their last menstrual period without any medical reason) and those who have undergone a hysterectomy and/or bilateral oophorectomy. Female patients who have been breastfeeding are eligible only if they have stopped breastfeeding before entry and agree to refrain from breastfeeding while receiving study treatment and for an additional week after the last infusion of tagraxofusp.

- 1. The patient has any other condition that disqualifies him/her from the study in the opinion of the investigator/sub-investigator.

**Tumor Response Criteria**

| Complete  Response  (CR) | Marrow*^a^ | Blast percentage ≤5% |
| --- | --- | --- |
|  | Peripheral blood | Neutrophil count ≥1,000/µL, platelet count ≥100,000/µL, and disappearance of blasts |
|  | Skin*^b^ | 100% clearance of all skin lesions from baseline (no new lesions in patients without lesions at baseline) |
|  | Nodal masses | Regression to normal size on CT |
|  | Spleen/liver | Not palpable, nodules disappeared |
| Complete Response with Incomplete Blood Count Recovery (CRi) | Marrow*^a^ | Blast percentage ≤5% |
|  | Peripheral blood | Incomplete recovery of neutrophil and/or platelet count(s) and absence of blasts |
|  | Skin*^b^ | 100% clearance of all skin lesions from baseline (no new lesions in patients without lesions at baseline) |
|  | Nodal masses | Regression to normal size on CT |
|  | Spleen/liver | Not palpable, nodules disappeared |
| CR with Minimal Residual Skin  Abnormality (CRc) | Marrow*^a^ | Blast percentage ≤5% |
|  | Peripheral blood | Neutrophil count ≥1,000/µL, platelet count ≥100,000/µL, and disappearance of blasts |
|  | Skin*^b^ | Marked clearance of all skin lesions from baseline, with residual BPDCN-related hyperpigmentation or abnormality identified by biopsy (or no biopsies available) |
|  | Nodal masses | Regression to normal size on CT |
|  | Spleen/liver | Not palpable, nodules disappeared |
| Partial Response (PR) | Marrow*^a^ | Decrease by ≥50% in blast percentage to 5–25% |
|  | Peripheral blood | Neutrophil count ≥1,000/µL and platelet count ≥100,000/µL |
|  | Skin*^b^ | 50% to <100% clearance of all skin lesions from baseline (no new lesions in patients without lesions at baseline) |
|  | Nodal masses | ≥50% decrease in the sum of the products of the greatest diameters (SPD) of index lesions (up to 6 largest dominant measurable masses); no increase in the size of other lymph node lesions |
|  | Spleen/liver | ≥50% decrease in the SPD of nodules (or in the greatest transverse diameter of a single nodule); no increase in size of liver or spleen |
| Stable Disease (SD) |  | Failure to achieve CR, CRi, CRc, or PR as defined above, but no evidence of progression for at least 8 weeks |
| Relapse after CR, CRi, or CRc*^c^ | Marrow*^a^ | Blast percentage ≥5% (if no peripheral blasts, then bone marrow aspirate is required for confirmation ≥1 week later) |
|  | Peripheral blood | Presence of blasts |
|  | Skin*^b^ | Increase in mSWAT skin score greater than the sum of nadir plus 50% baseline score |
|  | Nodal masses | Appearance of a new lesion(s) >1.5 cm in any axis, ≥50% increase from nadir in the SPD of more than one node, or ≥50% increase from nadir in the longest diameter of a previously identified node >1 cm in short axis |
|  | Spleen/liver | >50% increase from nadir in the SPD of any previous lesions |
| Relapse after PR*^c^ | Marrow*^a^ | Blast percentage ≥25% (if no peripheral blasts, then bone marrow aspirate is required for confirmation ≥1 week later) |
|  | Skin*^b^ | Increase in mSWAT skin score greater than the sum of nadir plus 50% baseline score |
|  | Nodal masses | Appearance of a new lesion(s) >1.5 cm in any axis, ≥50% increase from nadir in the SPD of more than one node, or ≥50% increase from nadir in the longest diameter of a previously identified lymph node lesion >1 cm in short axis |
|  | Spleen/liver | >50% increase from nadir in the SPD of any previous lesions |
| Progressive Disease (PD) *^c^ | Marrow*^a^ | ≥50% increase in blasts from baseline to >5% |
|  | Peripheral blood | One or more of the following:  - ≥50% decrease from peak response levels in platelets or granulocytes;  - Reduction in hemoglobin concentration by at least 2 g/dL;  - Transfusion dependence |
|  | Skin*^b^ | One or more of the following:  - ≥25% increase in mSWAT skin score from baseline;  - Any new tumors in patients without masses at baseline |
|  | Nodal masses | Appearance of a new lesion(s) >1.5 cm in any axis, ≥50% increase from nadir in the SPD of more than one node, or ≥50% increase from nadir in the longest diameter of a previously identified lymph node lesion >1 cm in short axis |
|  | Spleen/liver | >50% increase from nadir in the SPD of any previous lesions |

All parameters listed above (bone marrow, peripheral blood, skin [including quantification using mSWAT], lymph node, and visceral organs) were assessed both at baseline and at specified post-baseline time(s). Tumor response was determined by comparison with baseline or post-treatment nadir as specified above.

*a: Blast percentage was determined morphologically if any change in marrow blast percentage shown by flow cytometry was not consistent with that indicated by morphological assessment. Despite this, findings from flow cytometry were recorded.

*b: The extent of clearance or increase of skin lesions was calculated using the mSWAT.

*c: If there was preliminary, non-definitive evidence of disease progression (e.g., any unexplained new skin lesion, appearance of a node ≤1.5 cm, a slight increase after a marked decrease of marrow blast percentage, or appearance of a new blast population on flow cytometry not associated with such an increase of blast percentage that meets the criteria for PD), treatment with tagraxofusp could be continued only if the evidence of disease progression was not definitive and the documented conclusion reached by the investigator (subinvestigator) that continued treatment might be beneficial based on overall risk-benefit assessment. In addition, for patients whose response assessment of tagraxofusp differed between evaluation sites, tagraxofusp could be administered as an additional tagraxofusp infusion only if the investigator (subinvestigator) recorded that additional administration of tagraxofusp was desirable based on overall risk-benefit assessment. In all cases, relevant findings and assessments were recorded and lesions with signs of potential disease progression were further followed up.

**Definition of secondary efficacy endpoints**

OS was defined as the time from the date of the first tagraxofusp infusion until the date of death. The duration of CR+CRc was defined as the time from the first date a response (CR or CRc) was confirmed until the first date of an event (Relapse after CR or CRc, PR or SD after CR or CRc). The duration of the OR was defined as the time from the first date a response (CR, CRc, CRi, or PR) was confirmed until the first date of an event (Relapse after CR, CRc, or CRi, no longer failing to meet the criteria for a PR). PFS was defined as the time from the date of the first tagraxofusp infusion until the first date of an event (Death, PD). The duration of the BMCR was defined as the time from the date that BMCR was confirmed until the first date of an event (Relapse after CR or CRc or CRi, Relapse after PR, SD after achieving marrow remission, marrow blast percentage ≥5%).

**Definition of DLTs**

| - Grade 4 increased transaminase (AST/ALT) or CPK  - Grade 4^*a^ hematologic toxicity (not related to the underlying malignancy but to tagraxofusp) lasting for >21 days after the last infusion of tagraxofusp, or hematologic toxicity (not related to the underlying malignancy but to tagraxofusp) requiring treatment with hematopoietic factor (e.g., G-CSF) preparations  - Grade ≥3 non-hematologic toxicity (not related to the underlying malignancy but to tagraxofusp), except for a Grade 3 laboratory or clinical event (arthralgia, myalgia, pyrexia, diarrhea or nausea/vomiting^*b^) that recovers to Grade ≤1 or baseline within 21 days after the last infusion of tagraxofusp  - Any other toxicity (not related to the underlying malignancy but to tagraxofusp) that prevents the start of the next cycle of therapy within 14 days after the scheduled date |
| --- |

DLT, dose-limiting toxicity; AST, aspartate aminotransferase; ALT, alanine aminotransferase

The grade was determined according to CTCAE ver. 5.0.

*a: DLTs were determined by considering patient status, such as blood transfusion.

*b: Emetic episodes that require tube feeding, central vein alimentation, or hospital admission were regarded as DLTs.


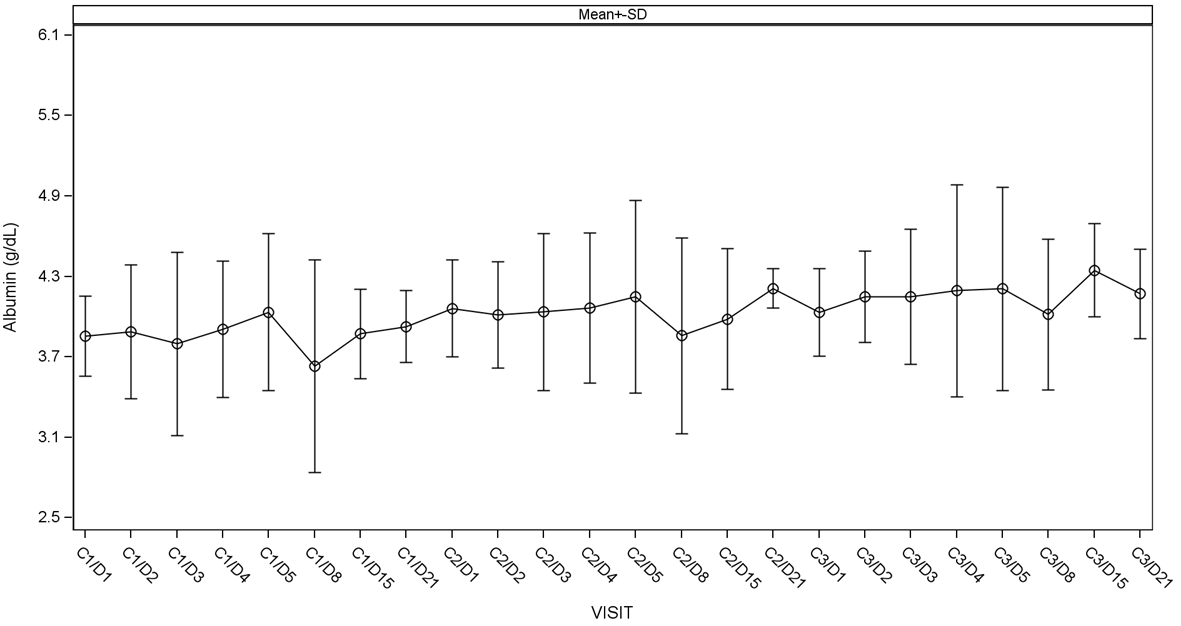
**Figures**

**Figure S1 Change in mean Albumin levels in all patients**


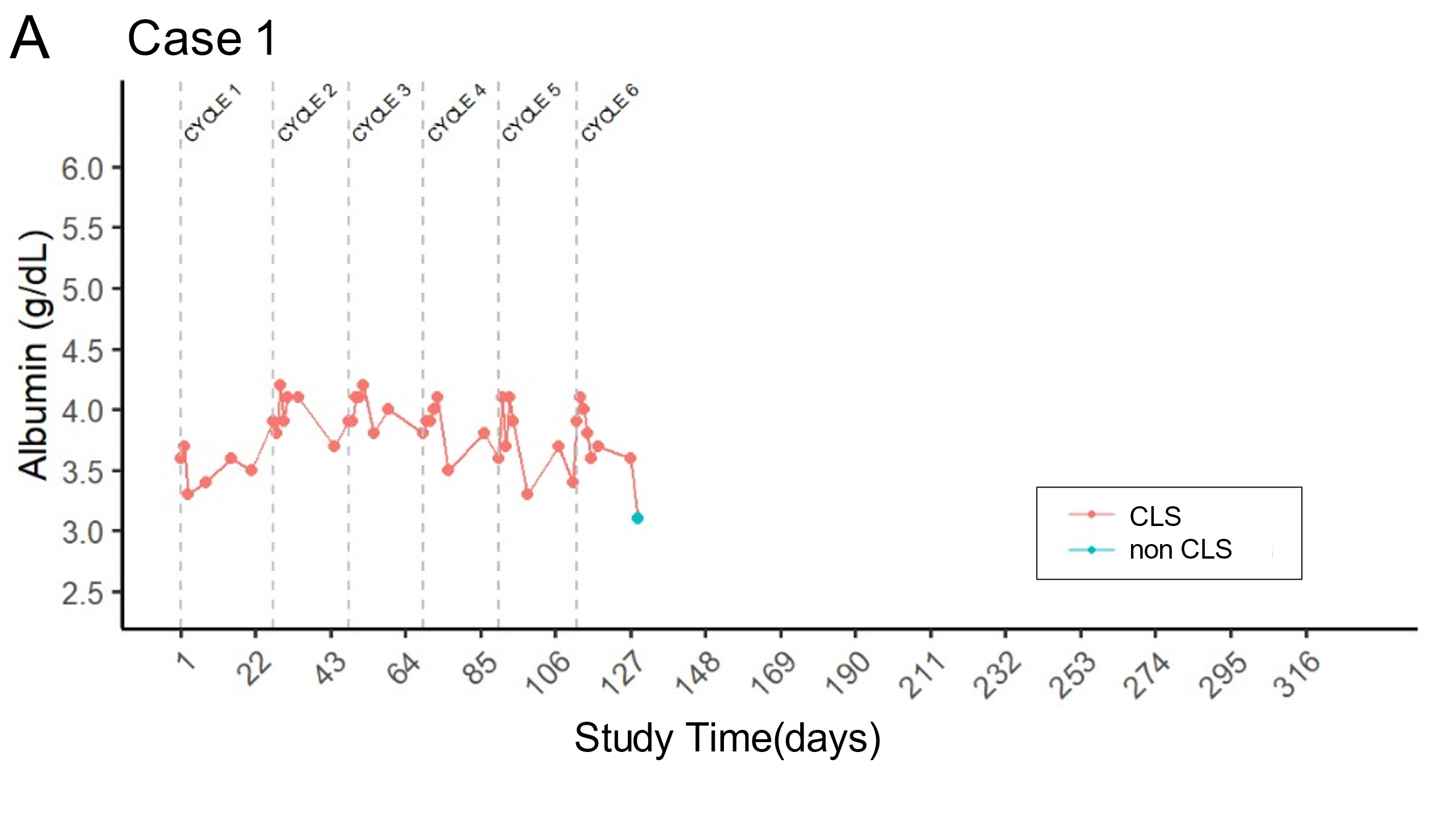

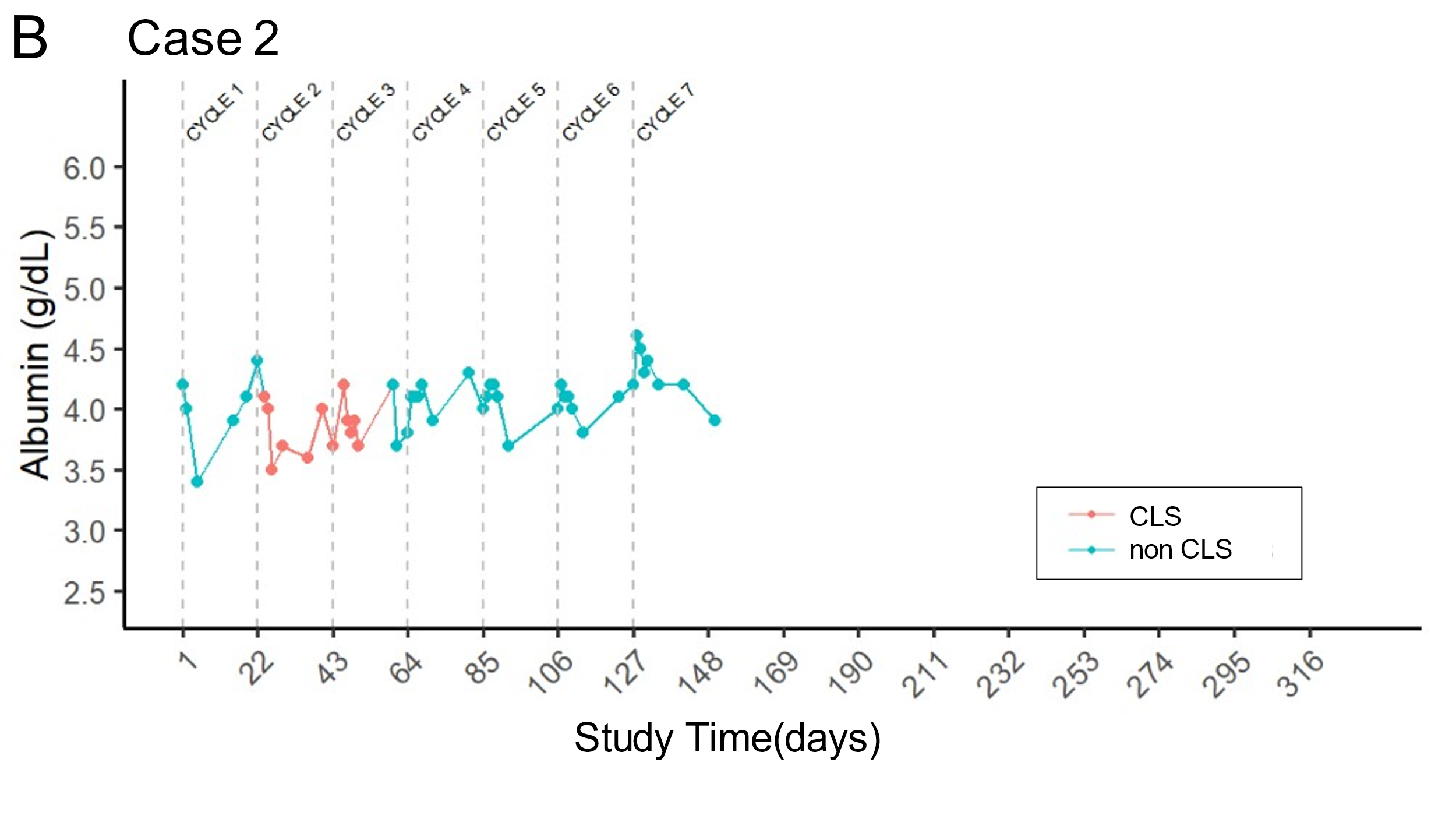

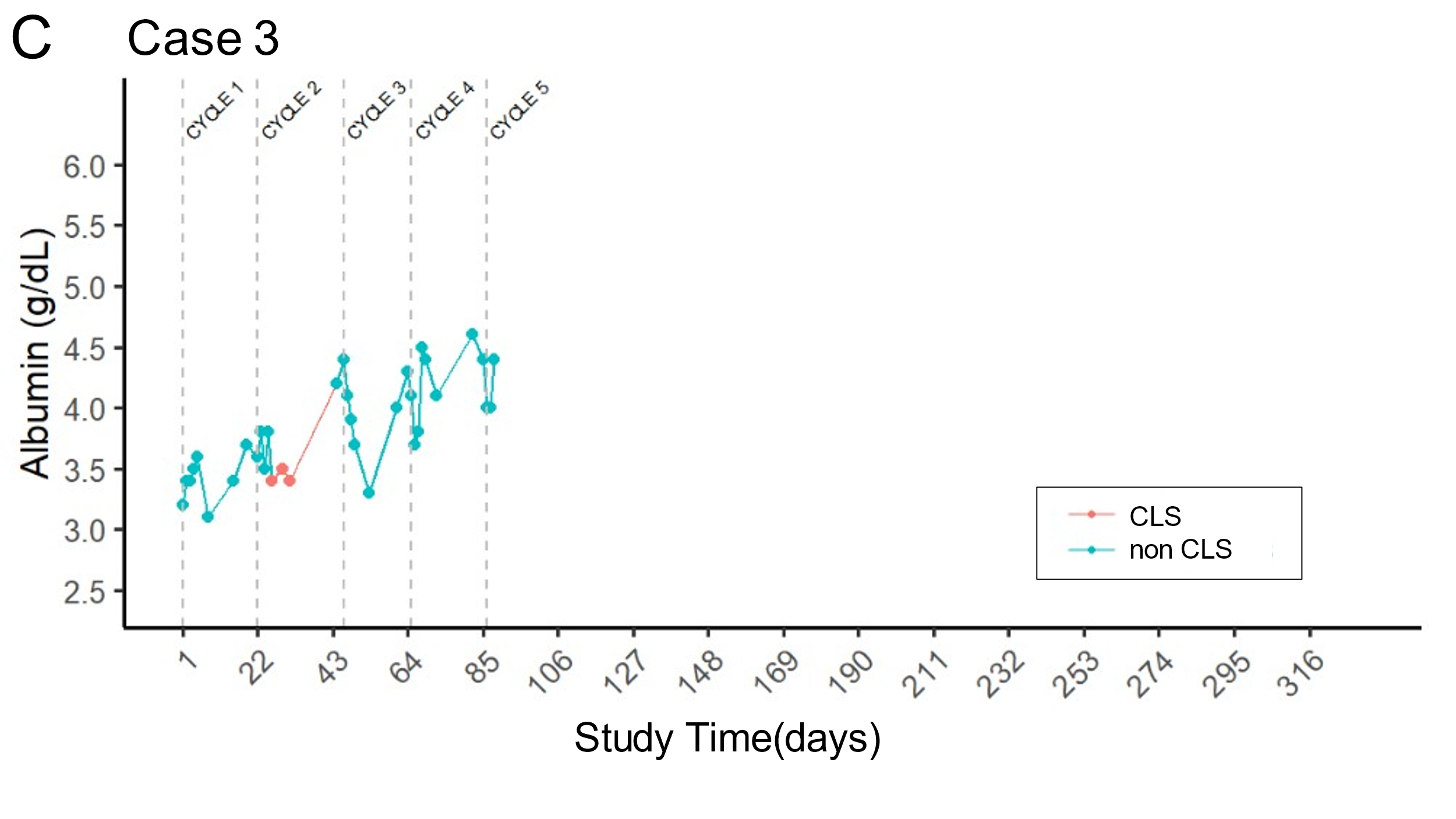

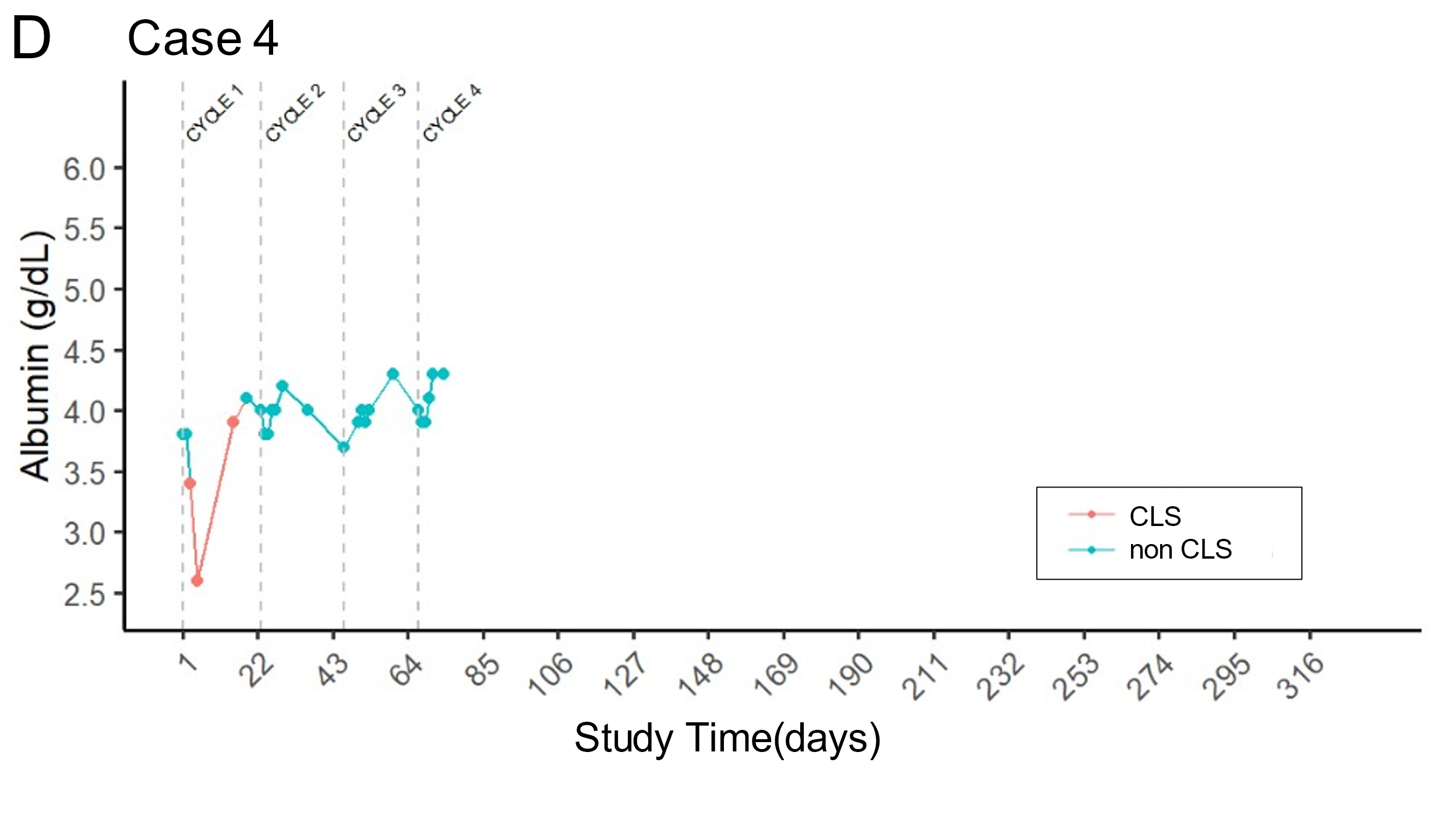

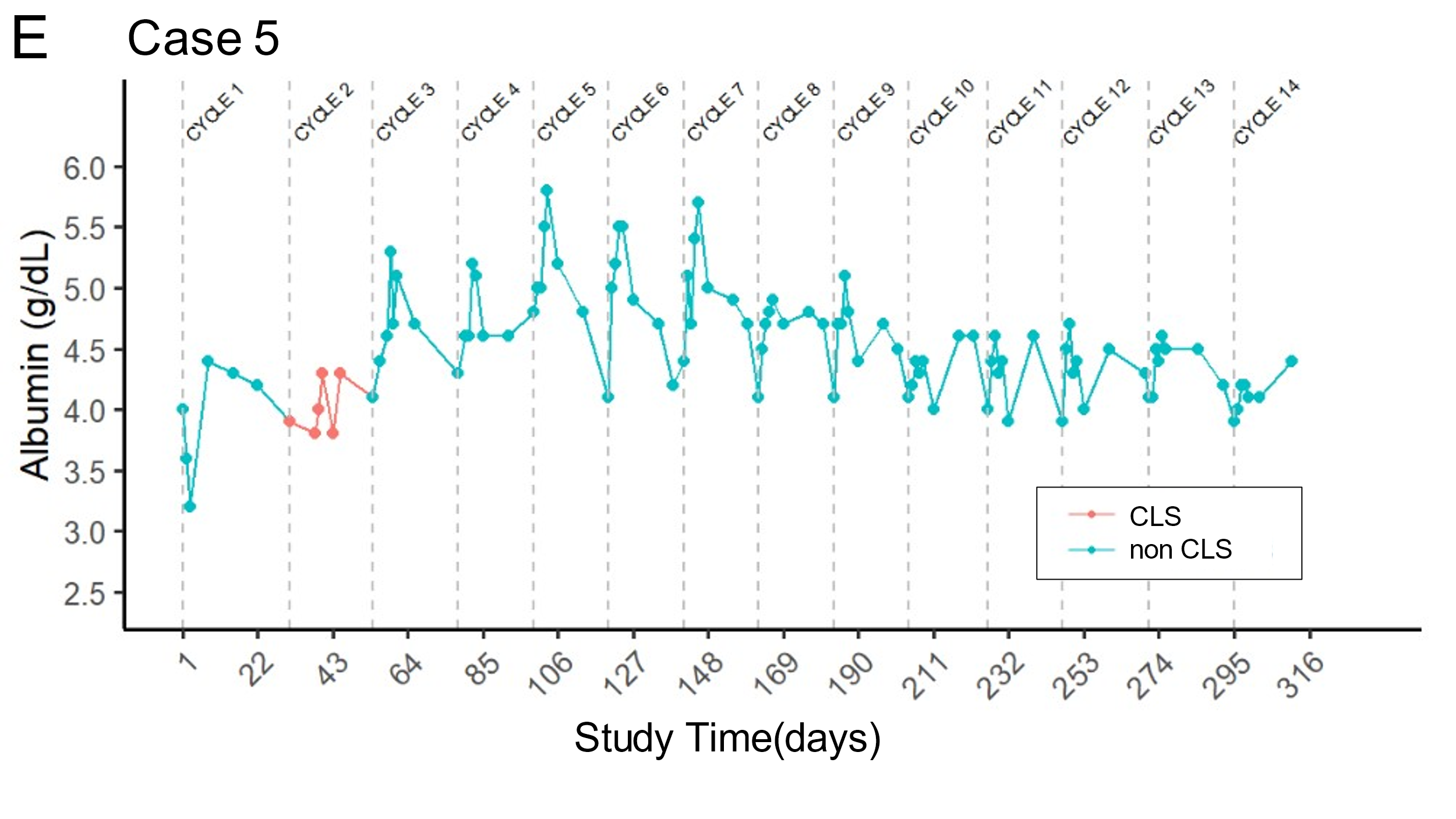

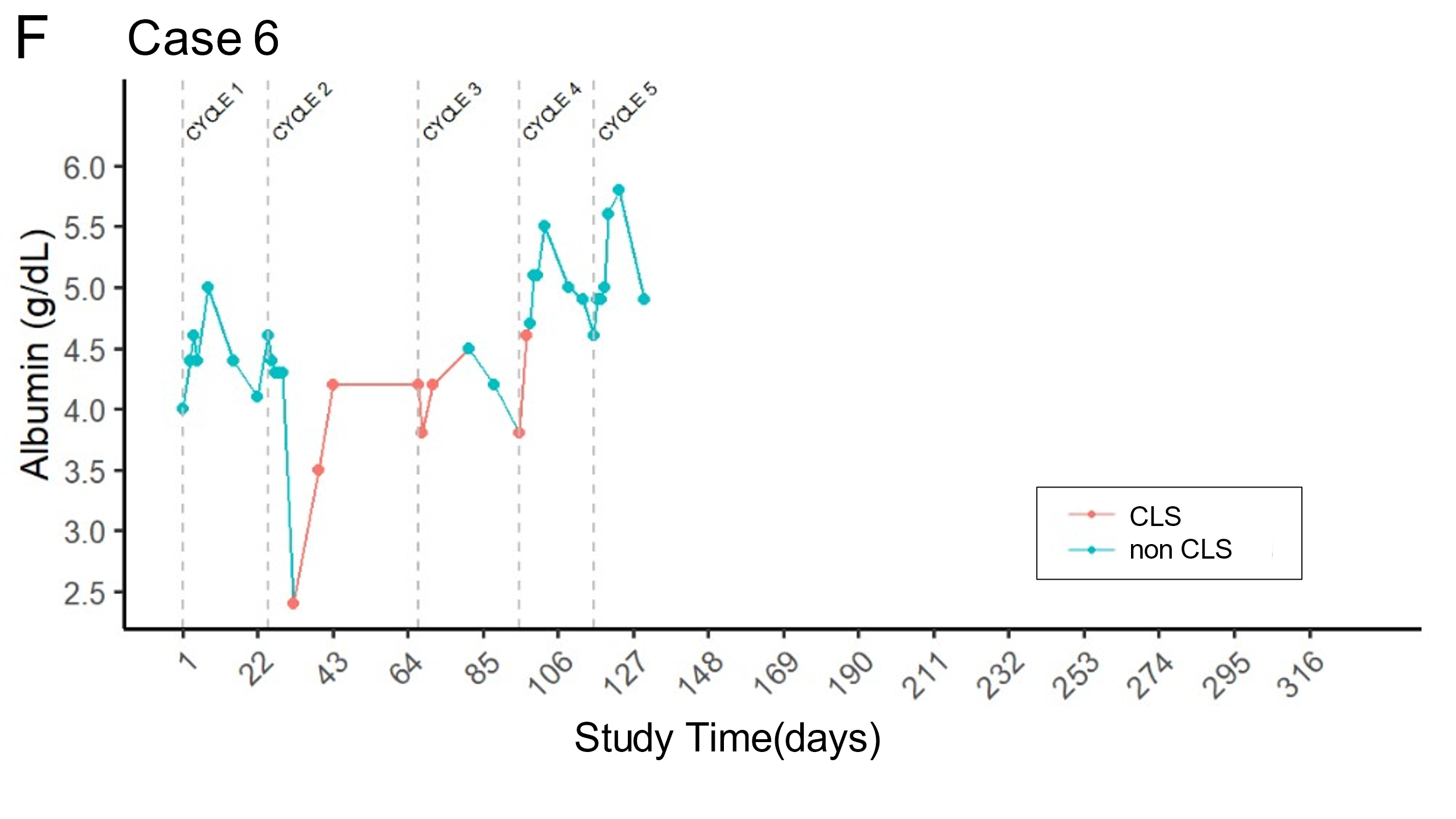


**Figure S2 Change in Albumin levels in patients with at least one occurrence of CLS (whole period)**

CLS, capillary leak syndrome


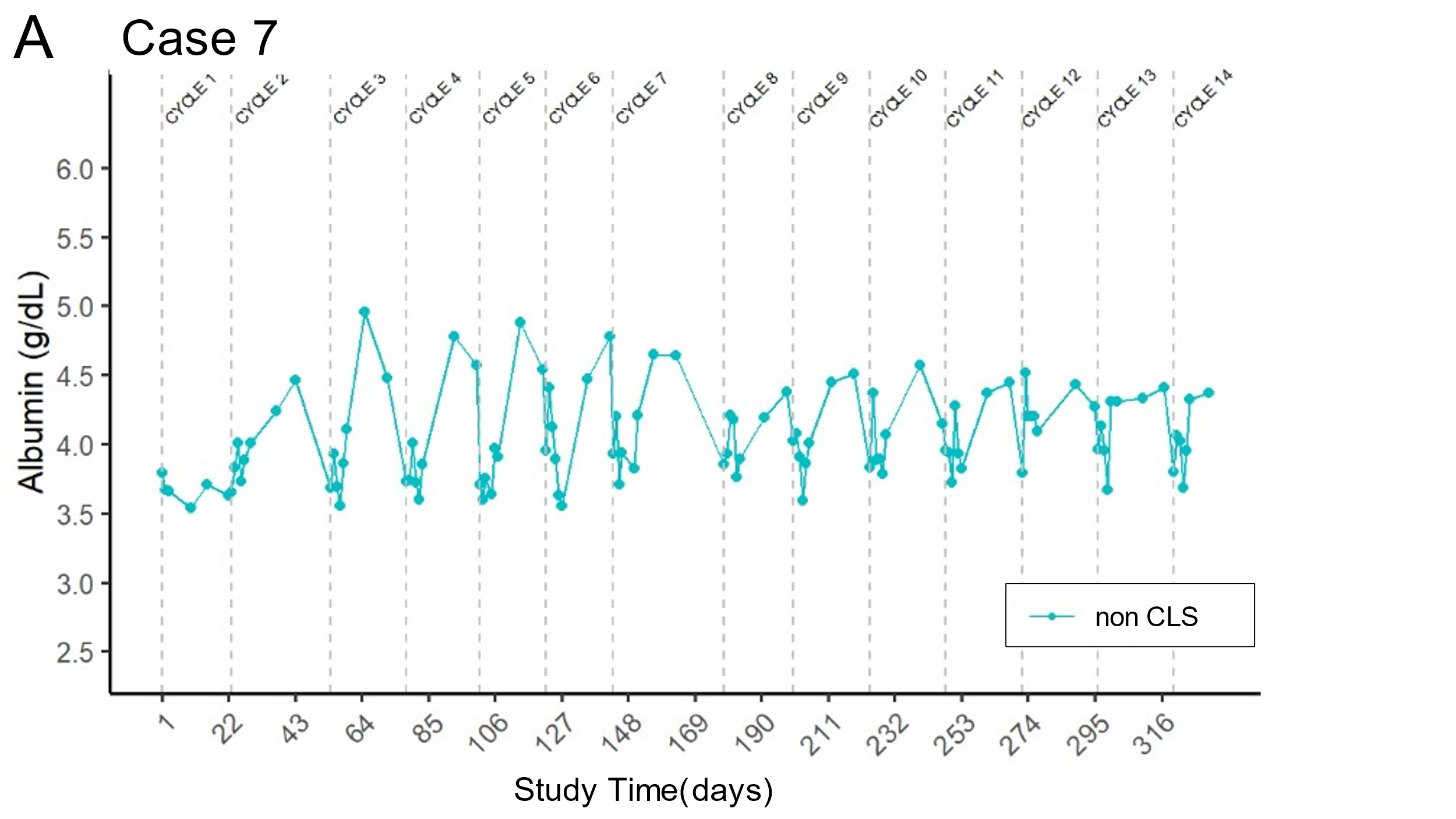

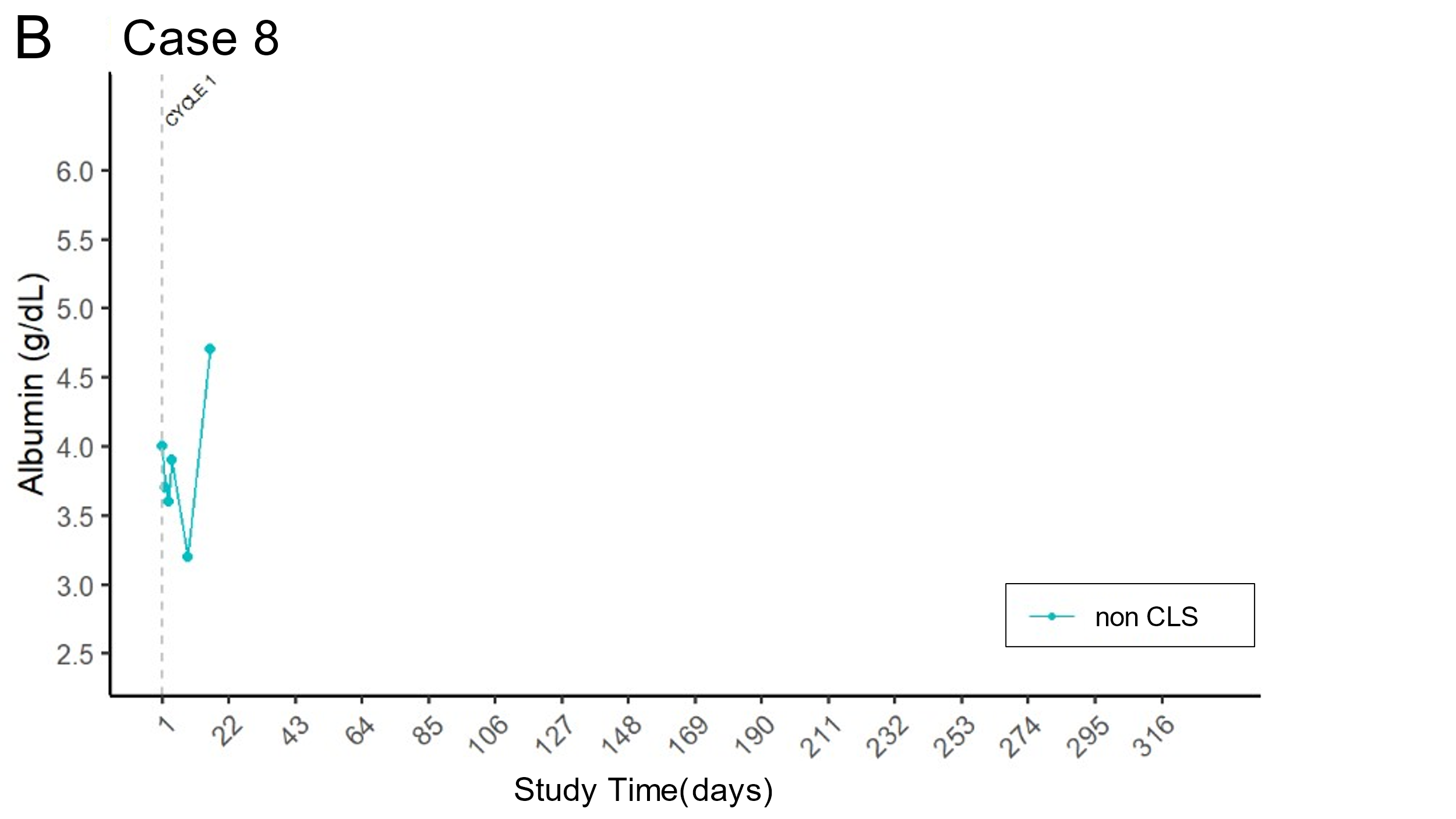

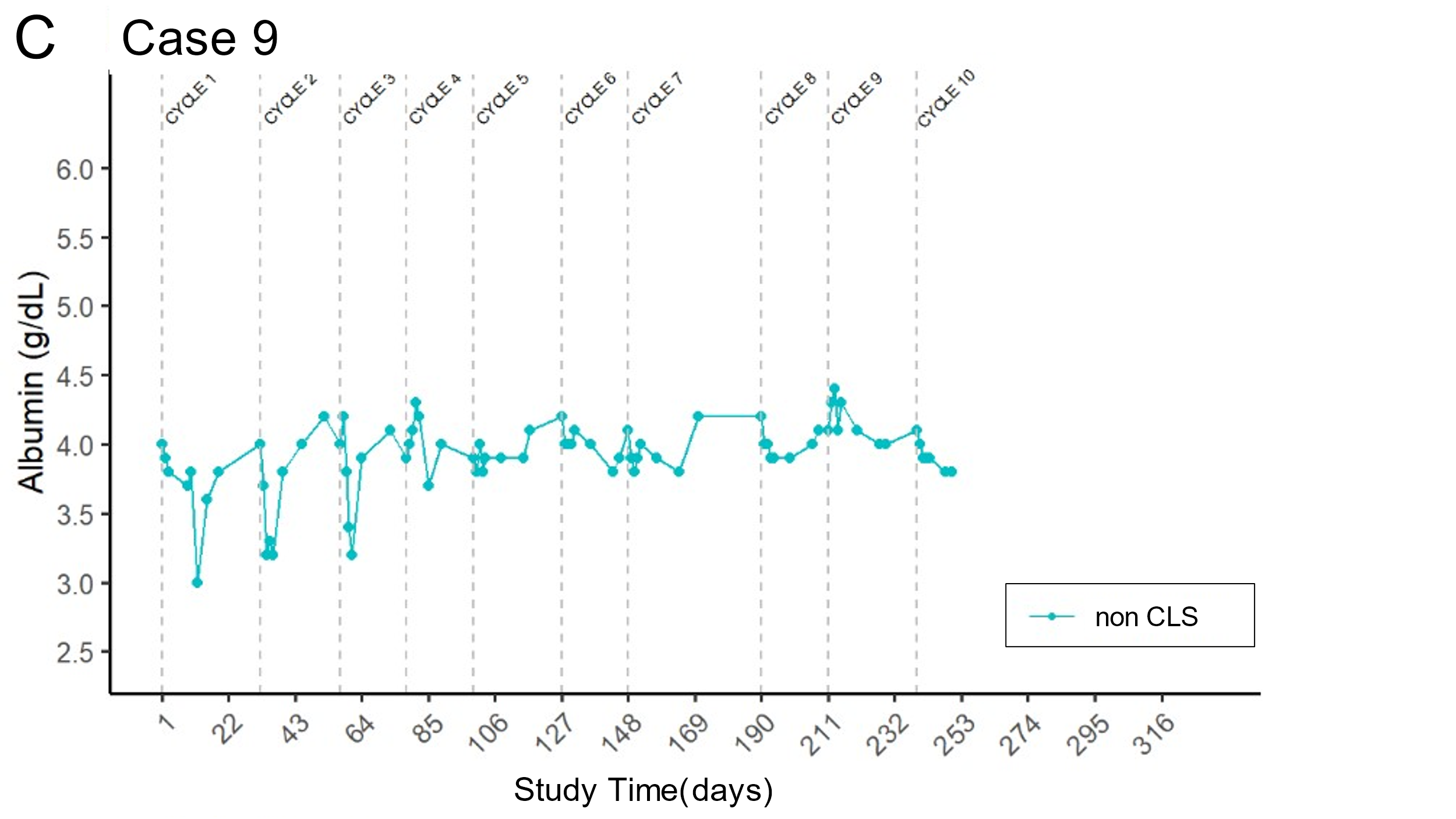

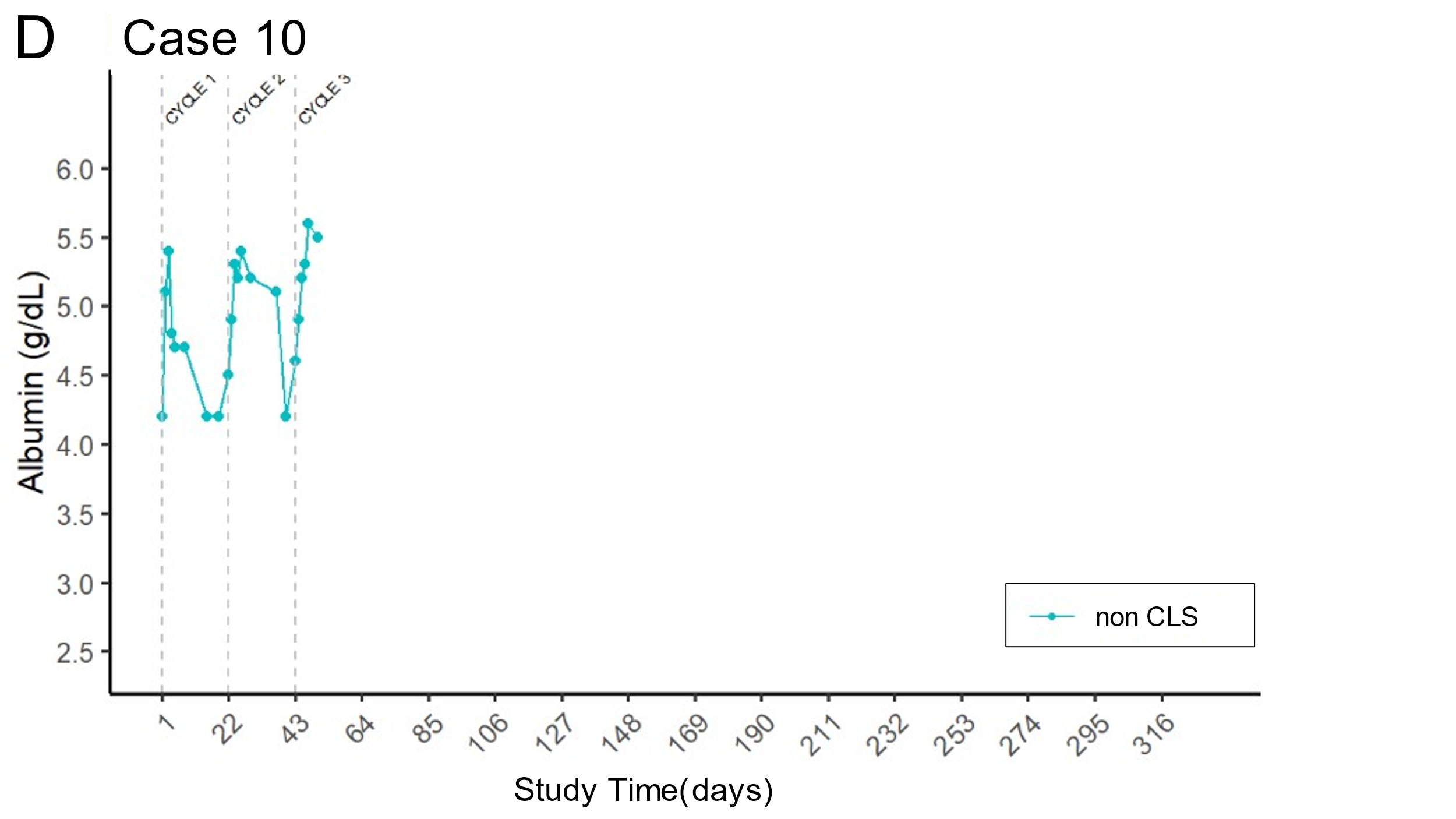

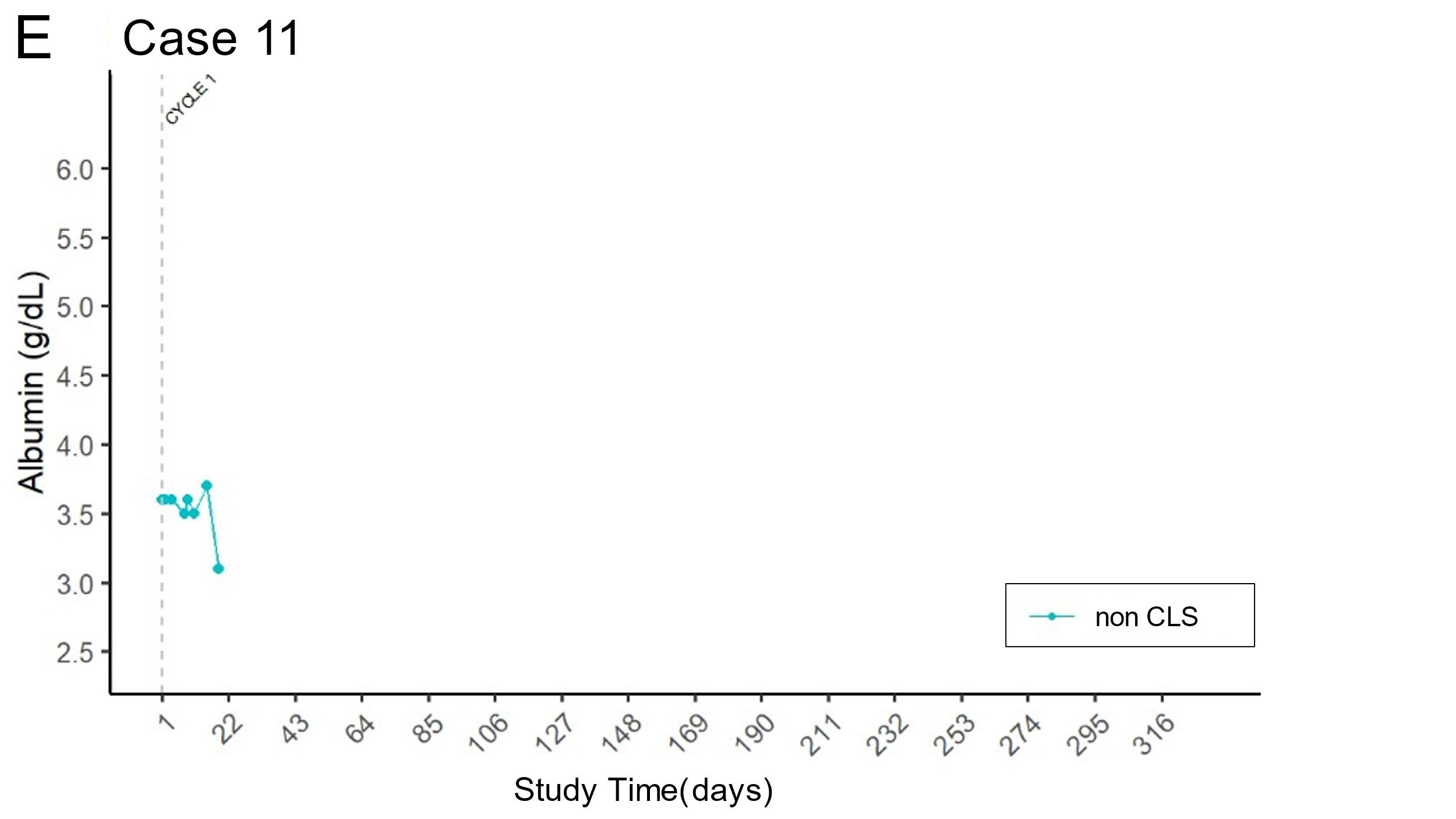


**Figure S3 Change in Albumin levels in patients without CLS (whole period)**

CLS, capillary leak syndrome


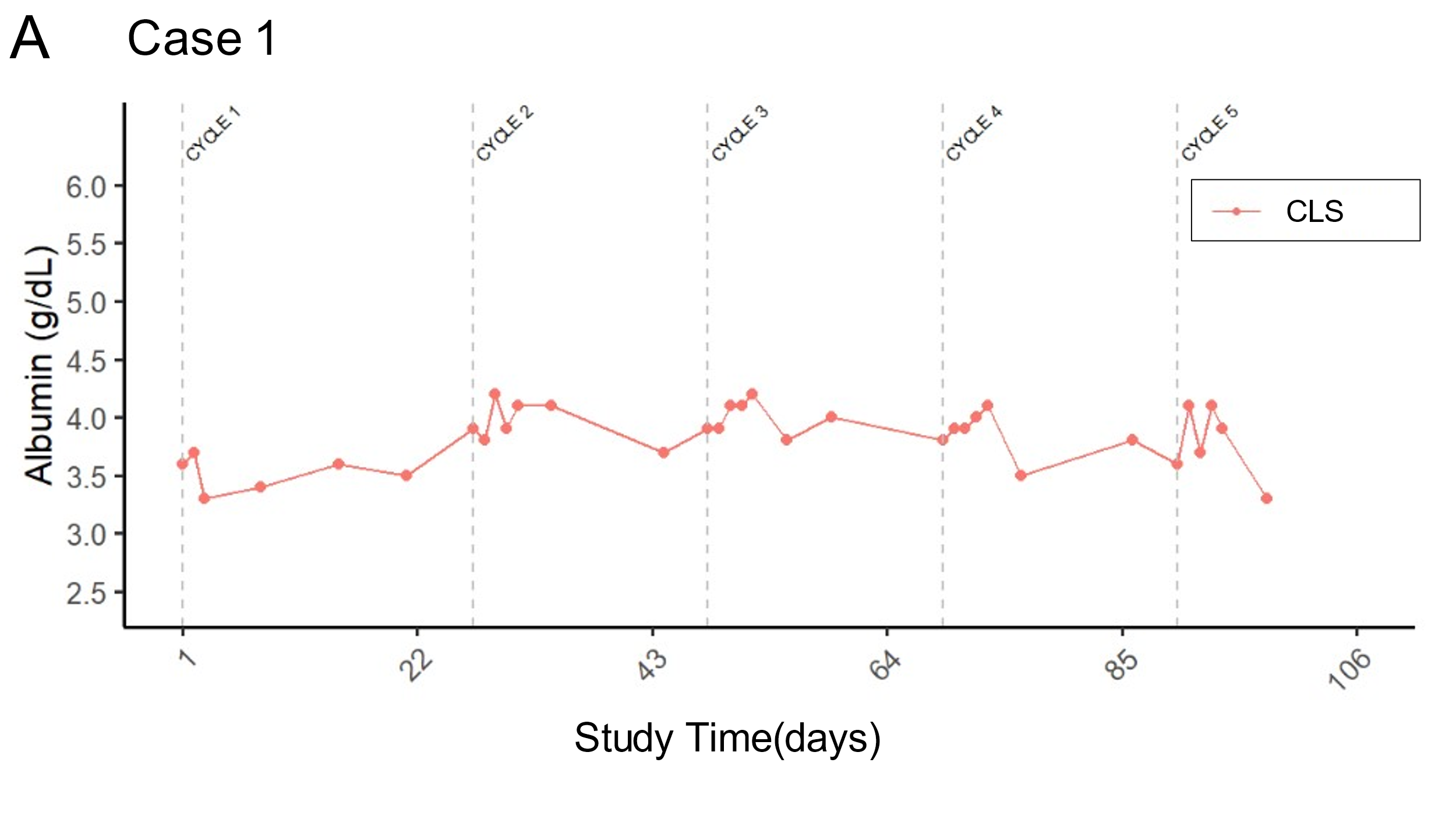

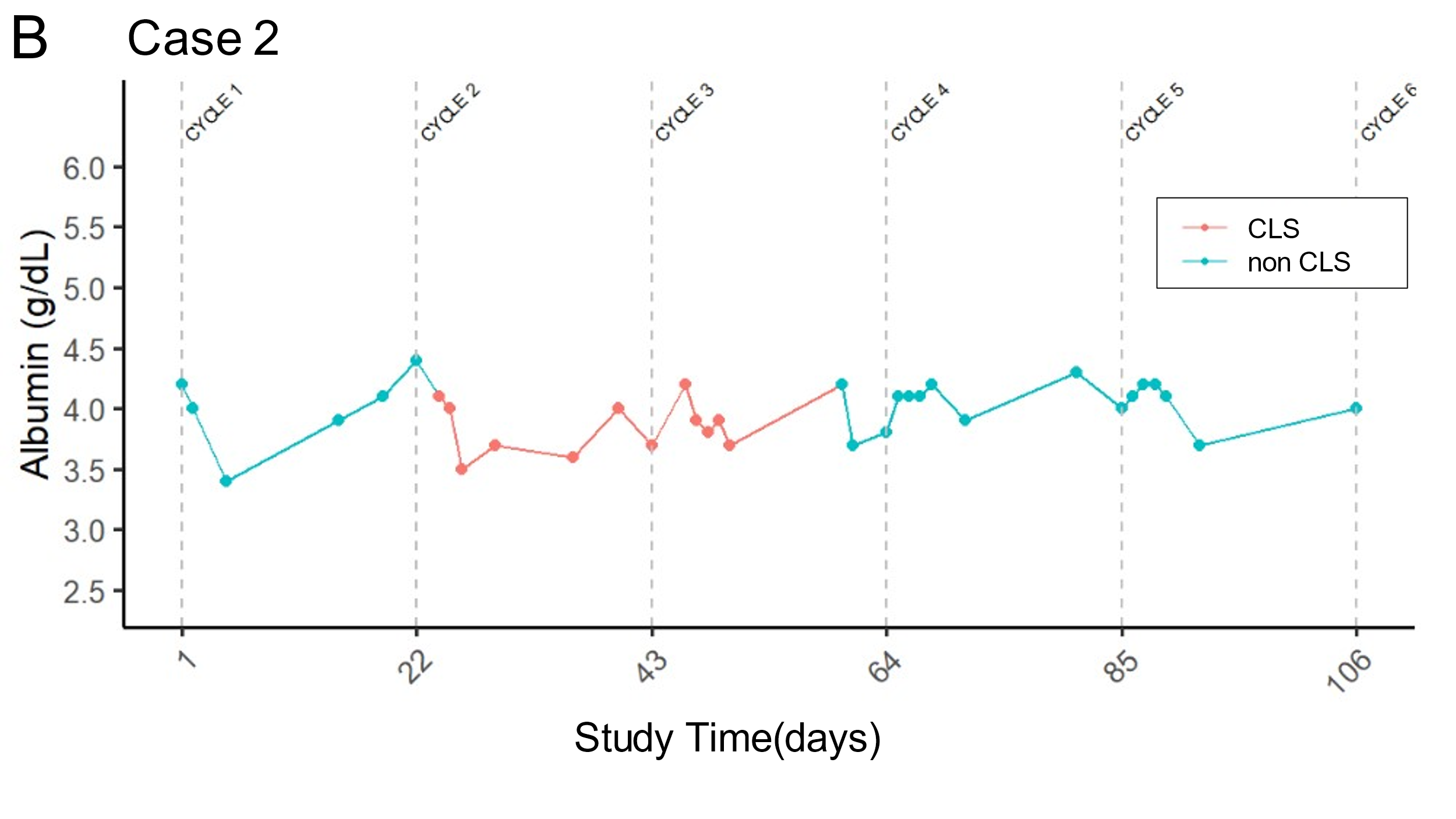

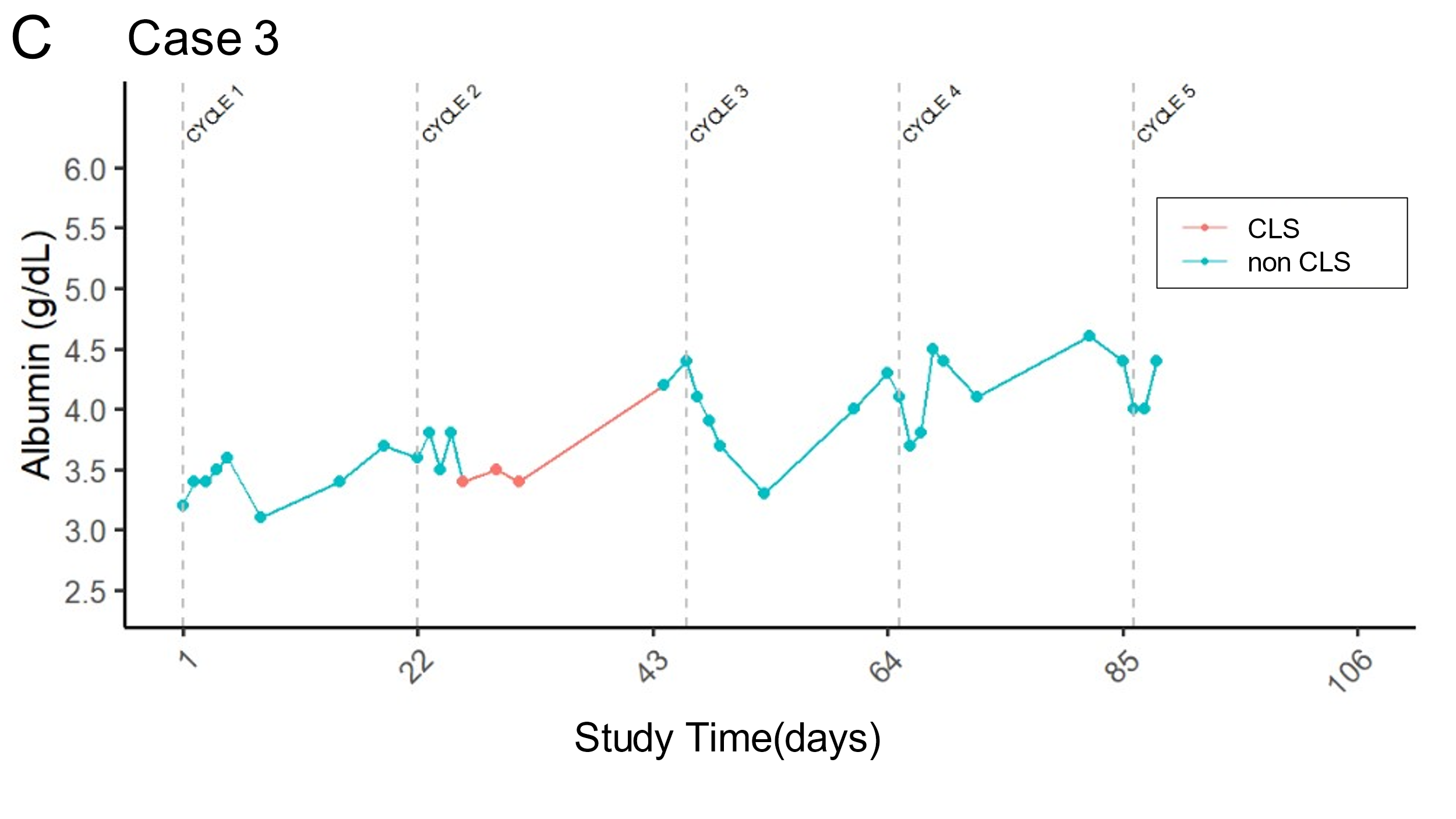

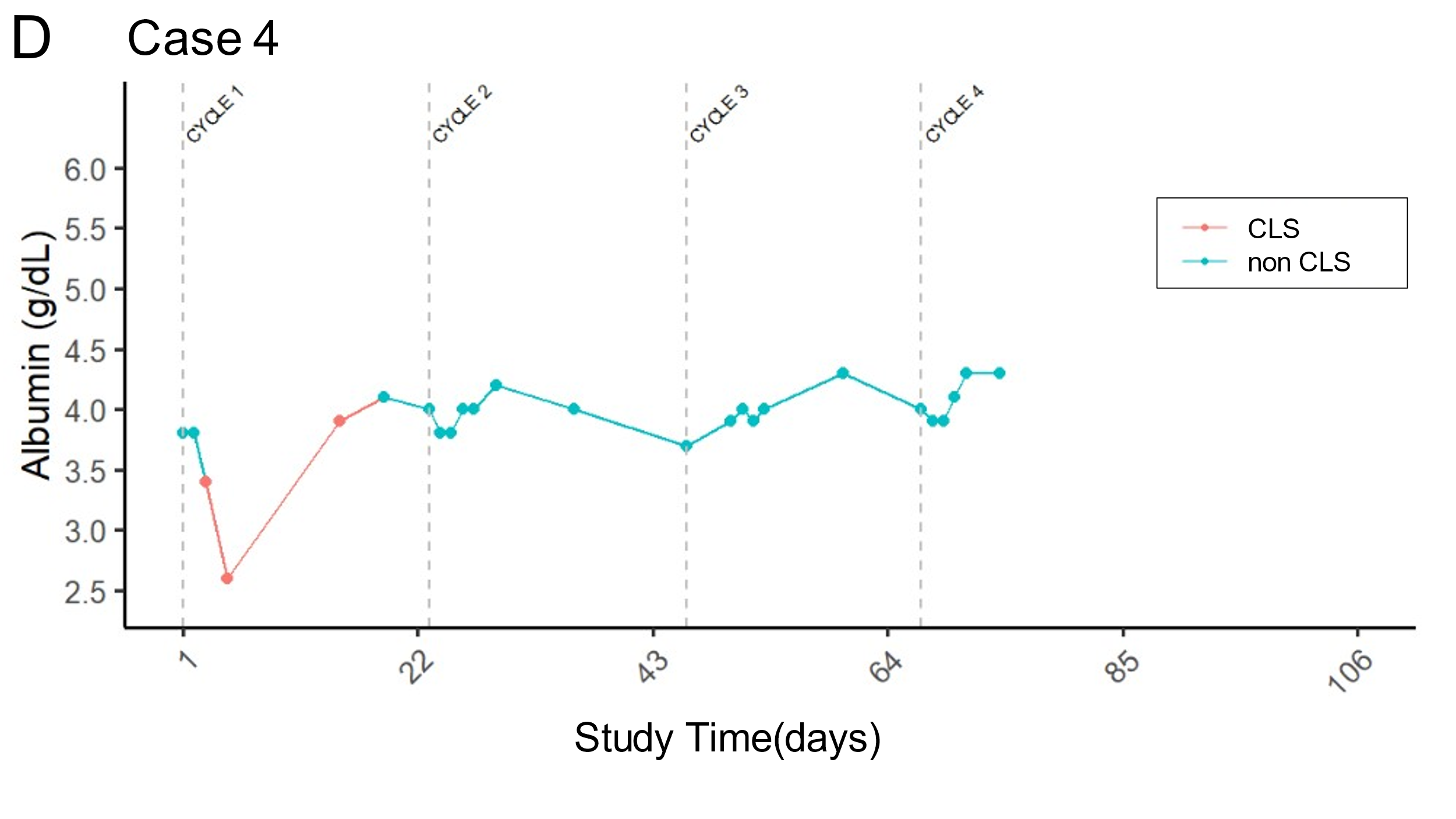

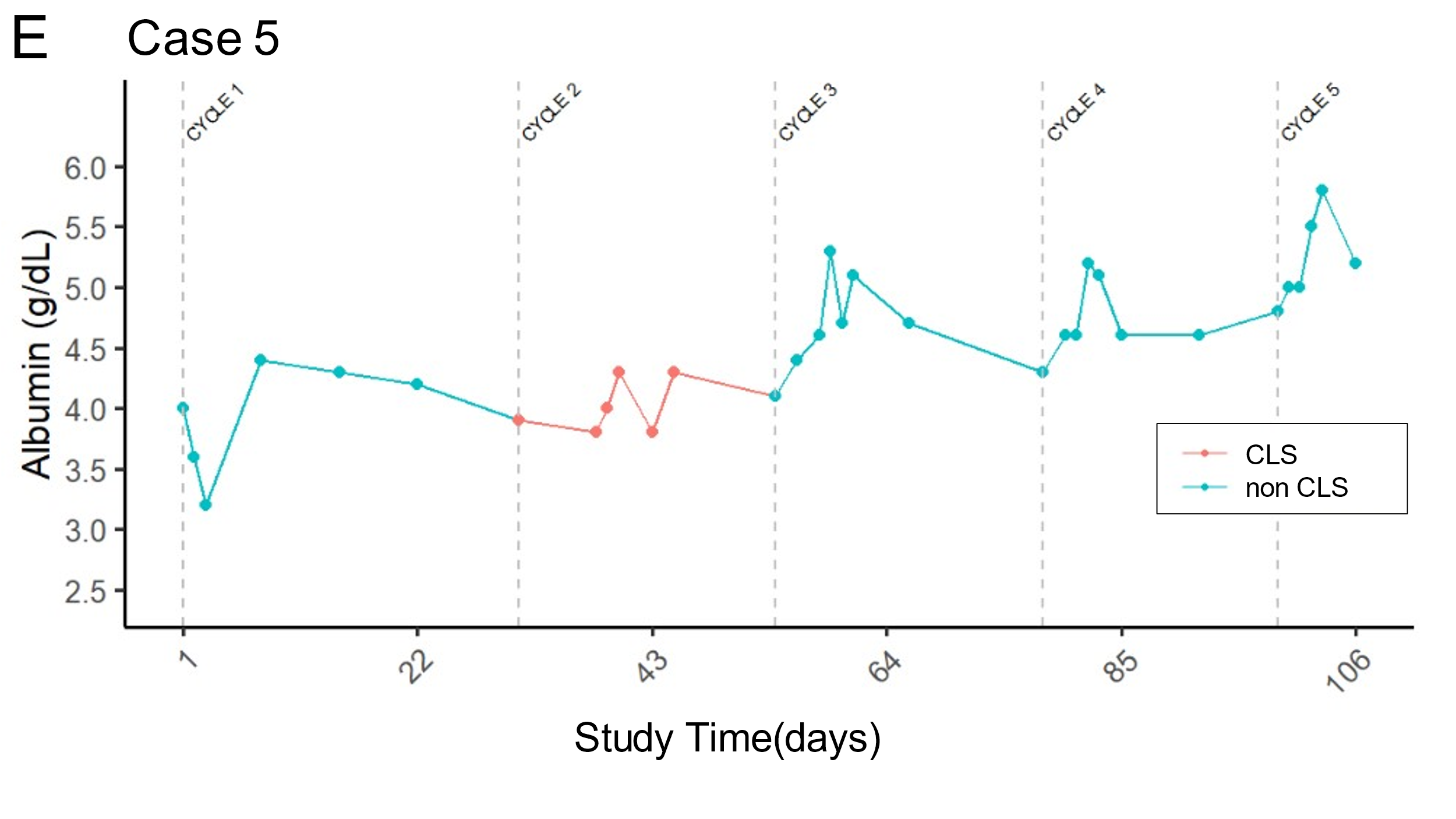

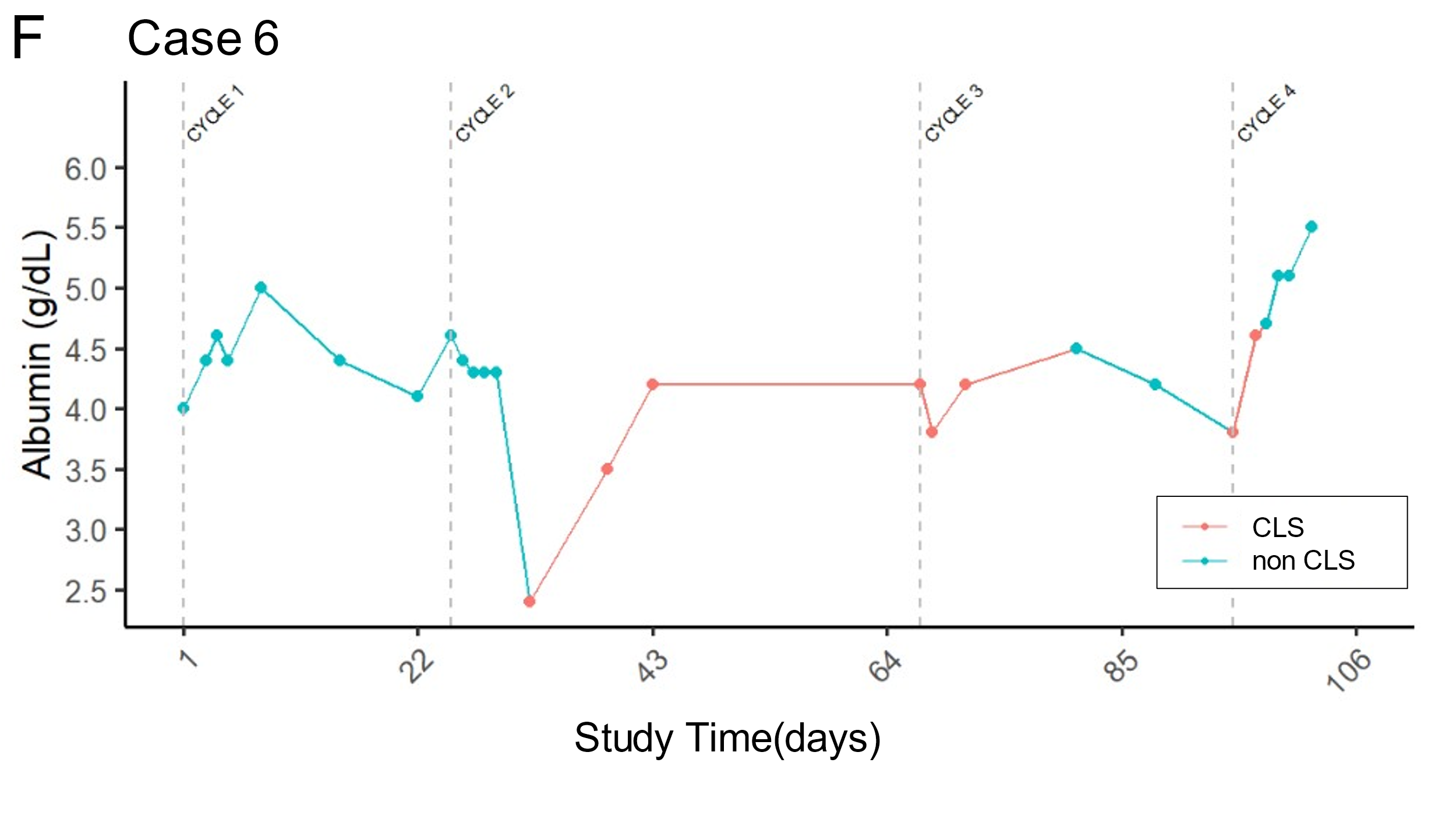


**Figure S4 Change in Albumin levels in patients with at least one occurrence of CLS (focus on early period）**

CLS, capillary leak syndrome


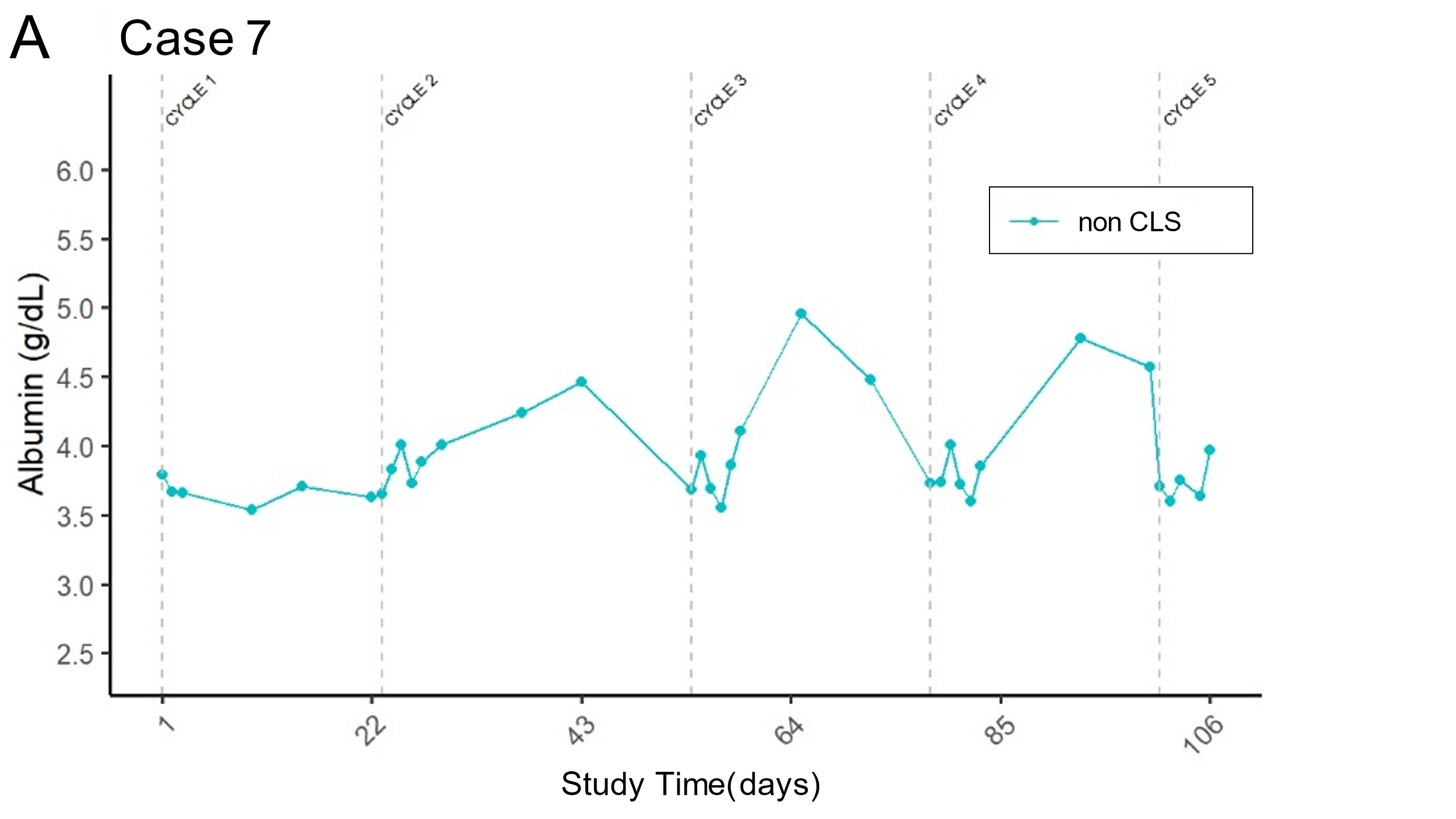

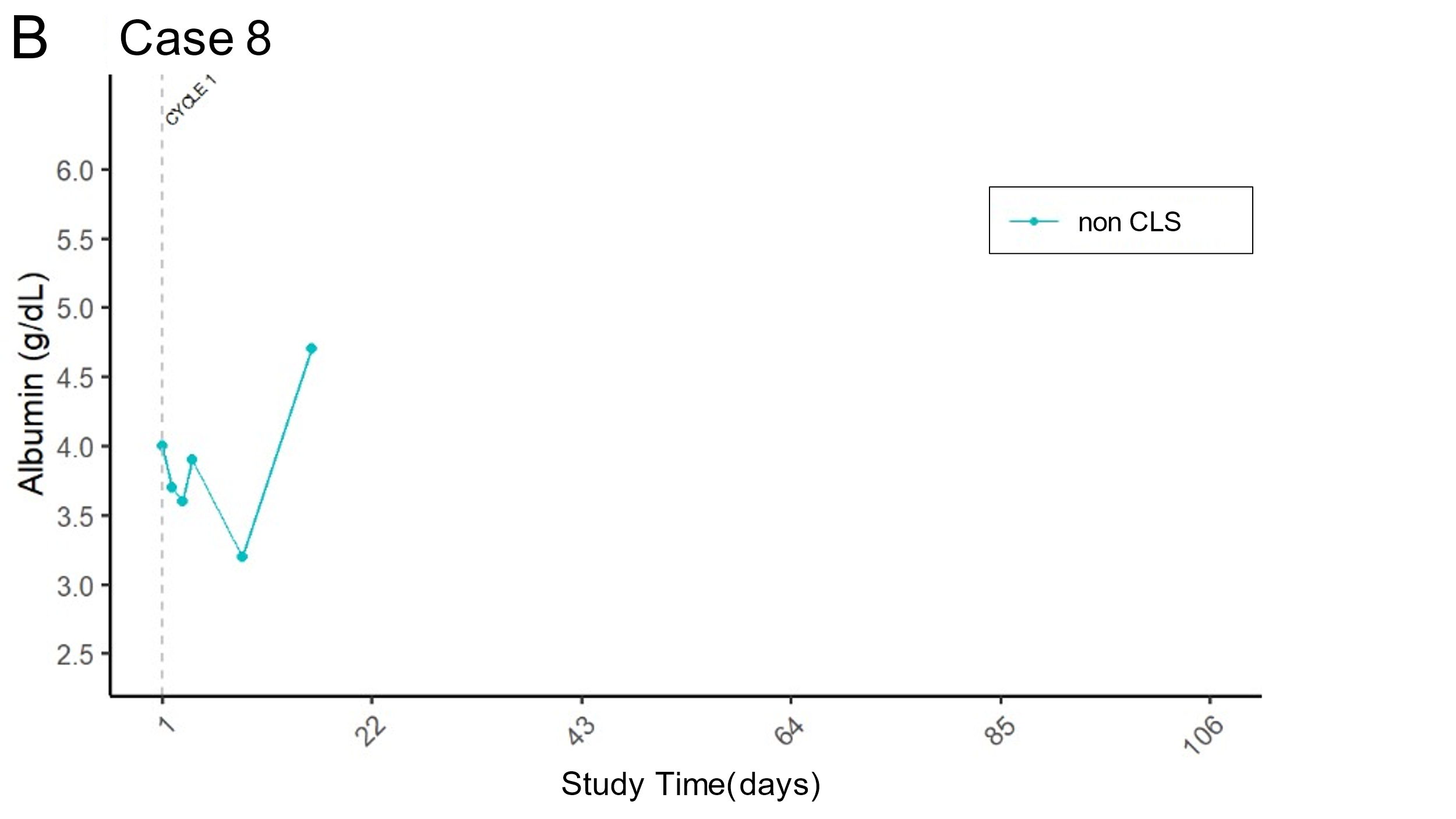

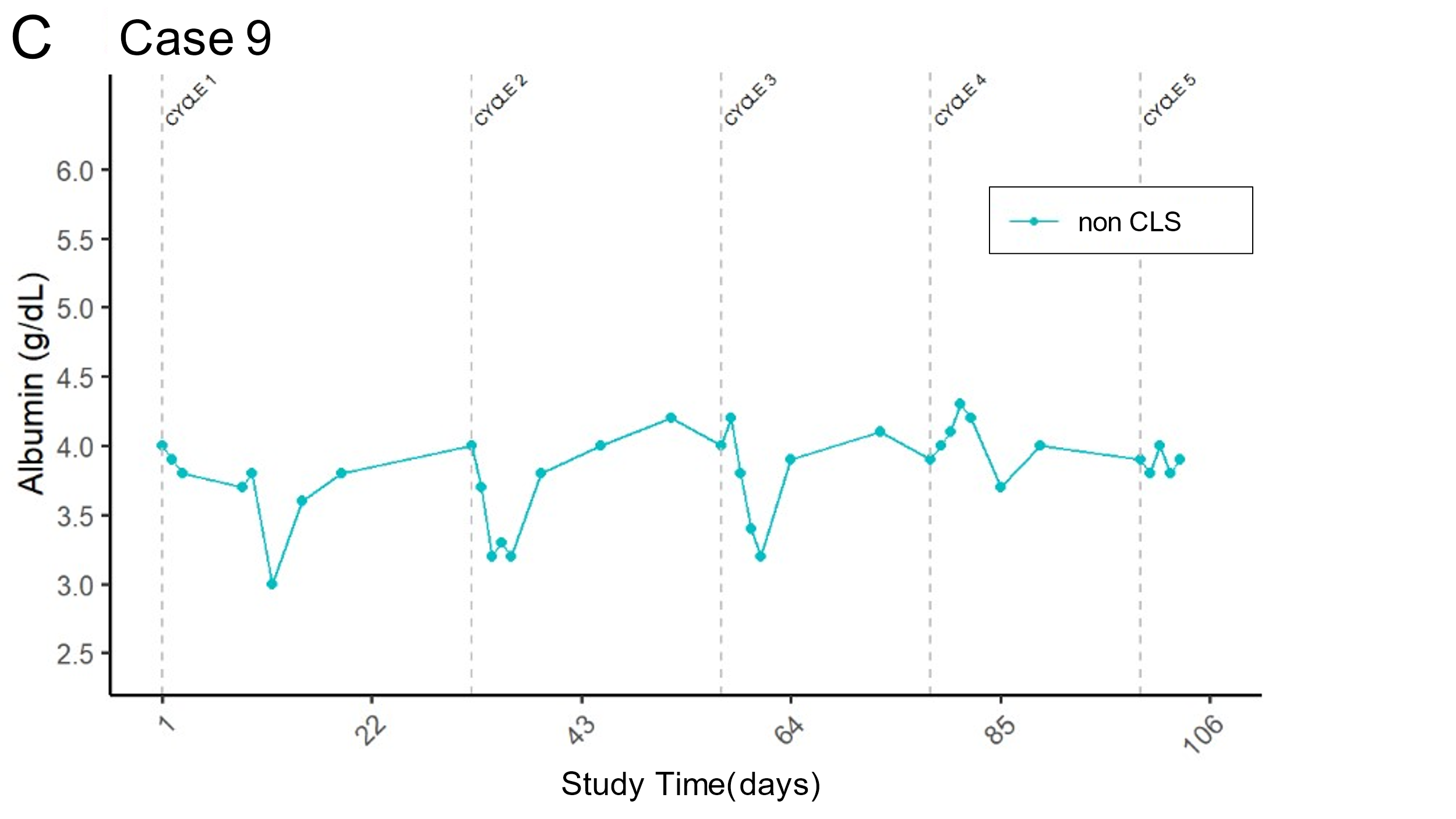

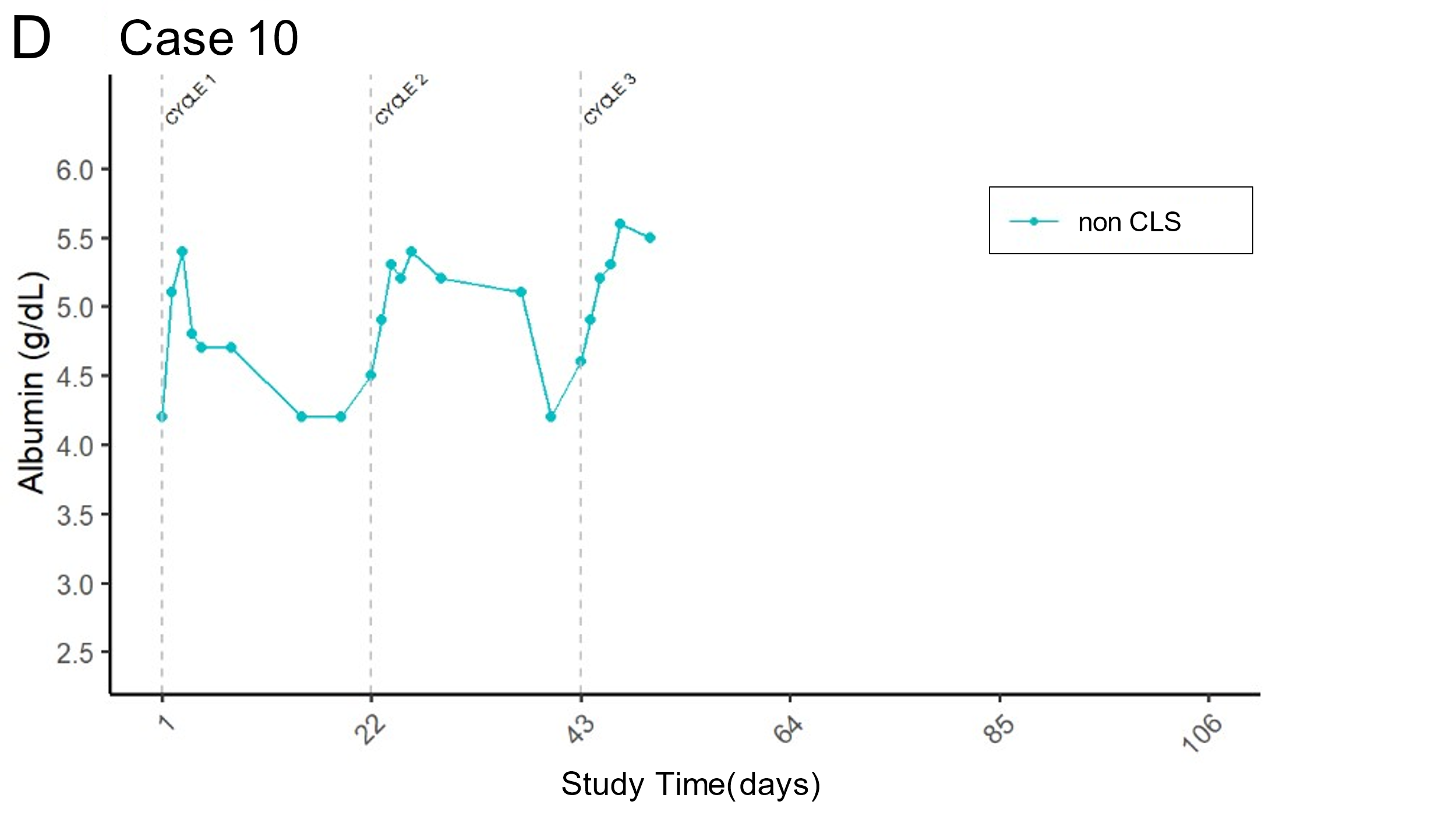

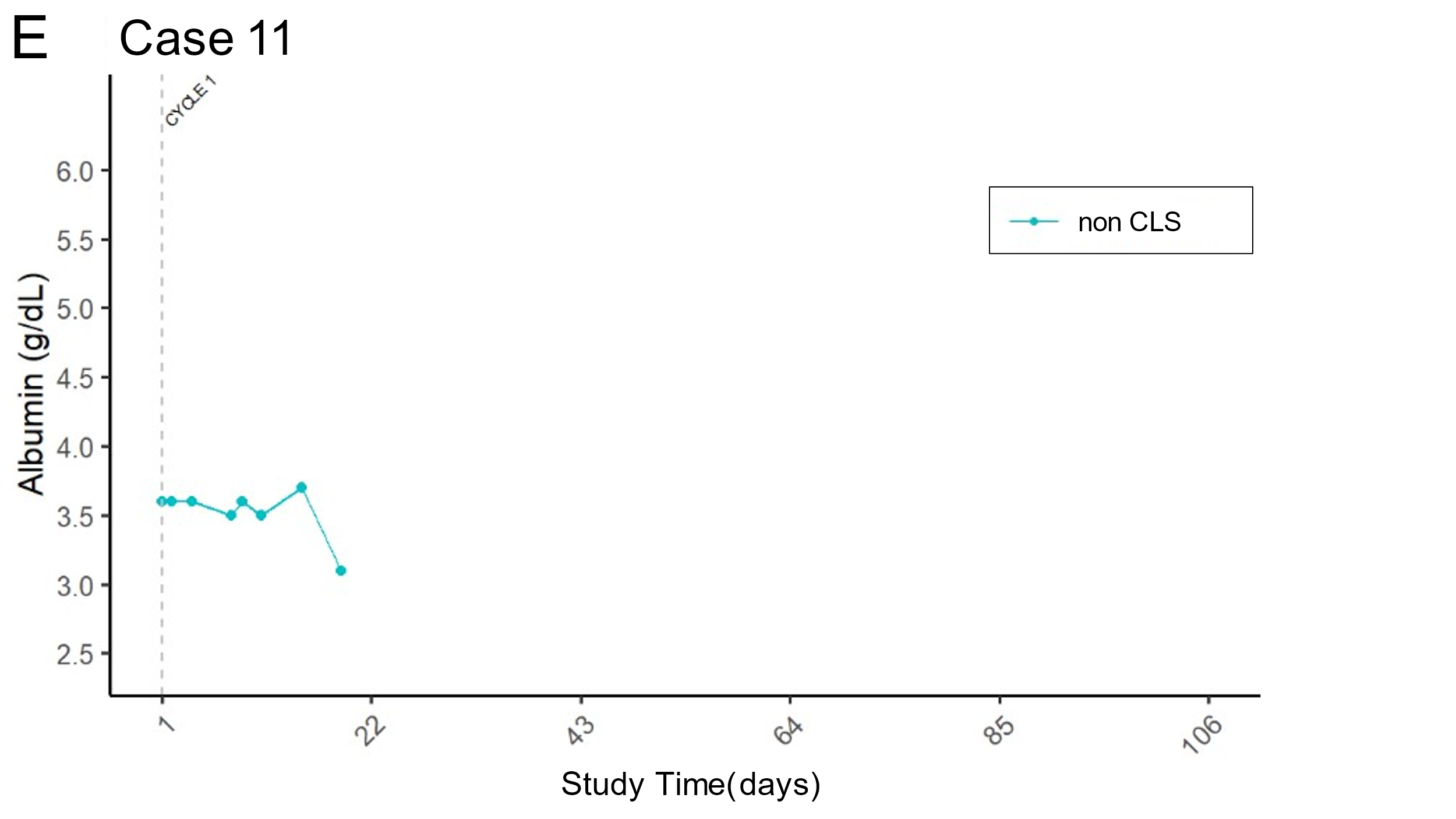


**Figure S5 Change in Albumin levels in patients without CLS (focus on early period）**

CLS, capillary leak syndrome

**
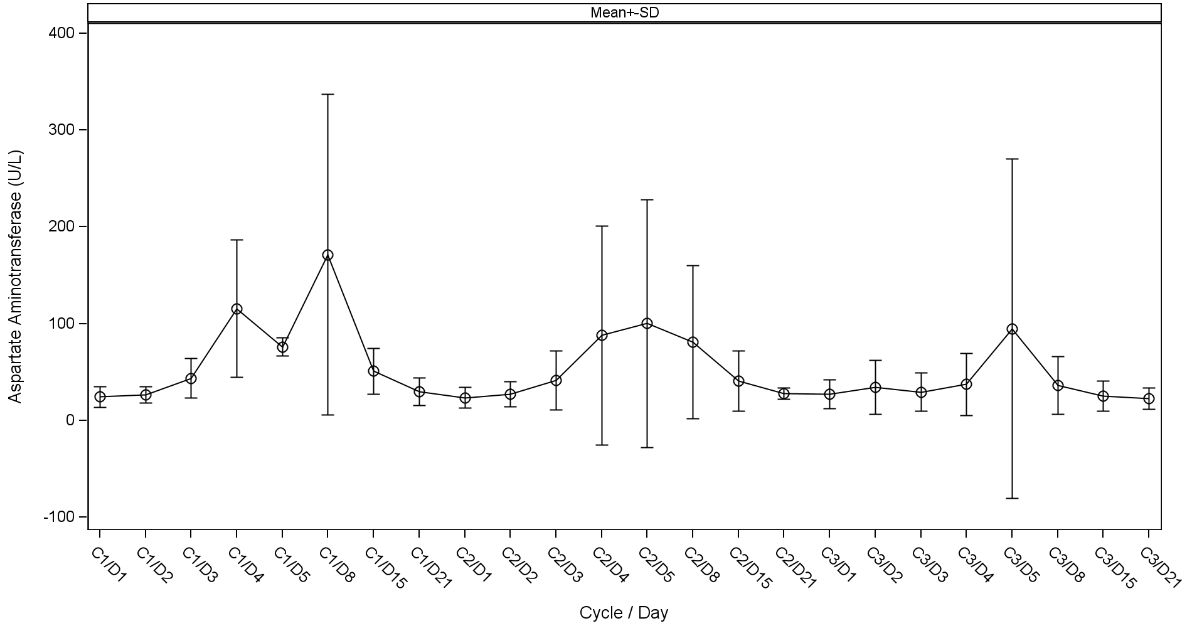
**

**Figure S6 Change in the mean aspartate aminotransferase levels in all patients**

**
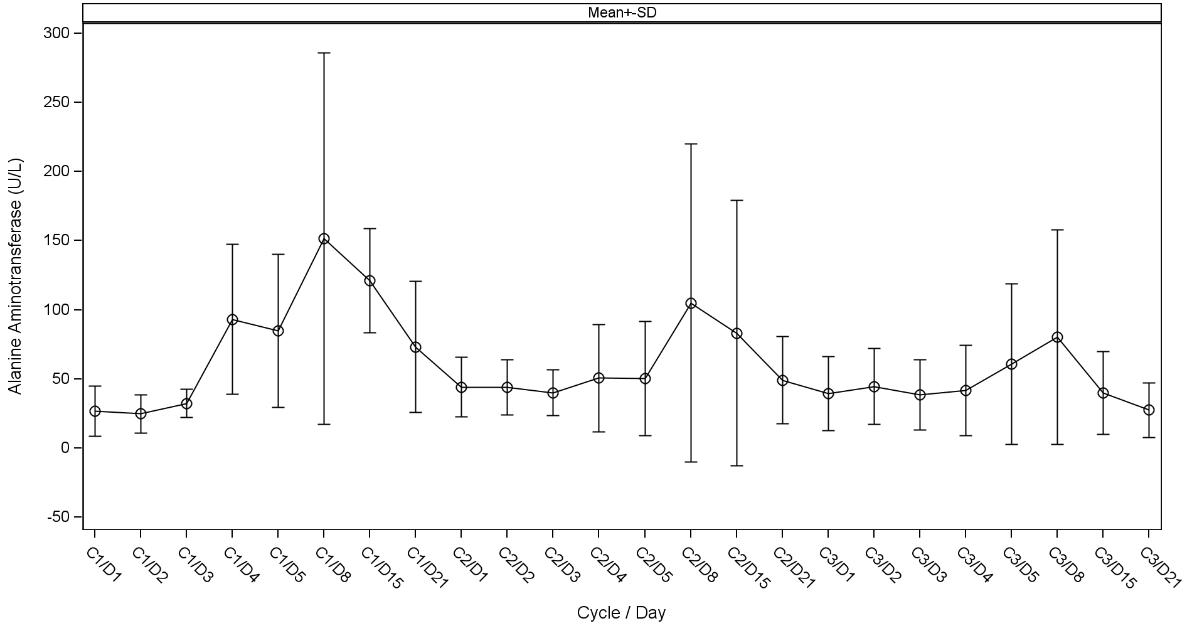
**

**Figure S7 Change in the mean alanine aminotransferase levels in all patients**

**Tables**

**Table S1 Prior therapies for BPDCN（FAS）**

|  | Relapsed/refractory  (N=4) |
| --- | --- |
| Prior therapies^*a^, n (%) |  |
| CHOP | 2 (50.0) |
| HD-MTX/AraC | 2 (50.0) |
| Radiation therapy | 2 (50.0) |
| R-CHOP | 1 (25.0) |
| CAR-T | 1 (25.0) |
| ESHAP | 1 (25.0) |
| Hyper-CVAD | 1 (25.0) |
| ICE | 1 (25.0) |
| modified MEAM | 1 (25.0) |
| Pola BR | 1 (25.0) |
| Auto-PBSCT | 1 (25.0) |

BPDCN, blastic plasmacytoid dendritic cell neoplasm; CHOP, cyclophosphamide, doxorubicin, vincristine, prednisone; HD-MTX/AraC, high dose methotrexate, cytarabine; CAR-T, chimeric antigen receptor-T cell; ESHAP, etoposide, methylprednisolone, cytarabine, cisplatin; Hyper-CVAD, cyclophosphamide, vincristine, doxorubicin, dexamethasone; ICE, ifosfamide, cisplatin, etoposide; modified MEAM, ranimustine, cytarabine, melphalan, vindesine sulfate; Pola BR, polatuzumab vedotin, bendamustine, rituximab; R-CHOP, rituximab, cyclophosphamide, doxorubicin, vincristine, prednisone; PBSCT, peripheral blood stem cell transplantation

*a: Patients may have received multiple prior therapies, so percentages do not sum to 100% and total N is not applicable to sum of events.

**Table S~~1~~2 Secondary efficacy endpoints（FAS）**

|  | Treatment-naïve  (N=7) | Relapsed/refractory  (N=4) |
| --- | --- | --- |
| Duration of CR＋CRc（months） |  |  |
| Median  90%CI | NA  NA | NA  NA |
| OS（months） |  |  |
| Median  90%CI | NA  5.67, NA | 6.67  5.53, NA |
| Duration of OR（months） |  |  |
| Median  90%CI | NA  2.83, NA | NA  2.60, NA |
| BMCR | 6 | 4 |
| n (%)  90%CI | 4 (66.7)  27.13, 93.72 | 2 (50.0)  9.76, 90.24 |
| Duration of BMCR（months） |  |  |
| Median  90%CI | NA  2.77, NA | NA  1.37, NA |
| Percentage of patients undergoing HSCT |  |  |
| n (%)  90%CI | 1 (14.3)  0.73, 52.07 | 1 (25.0)  1.27, 75.14 |
| PFS（months） |  |  |
| Median  90%CI | NA  1.50, NA | NA  0.53, NA |

CR, complete remission; CRc, complete remission with minimal residual skin abnormality; OS, overall survival; OR, overall response (CR+CRc+complete response with incomplete blood count recovery (CRi) + partial remission (PR)); ORR, overall response rate; BMCR, bone marrow complete response; HSCT, hematopoietic stem cell transplantation; PFS, hematopoietic stem cell transplantation; NA, not applicable

**Table S~~2~~3 Incidence of adverse events related to capillary leak syndrome (SAF)**

|  | Adverse event  (any grade) | Adverse  drug reaction  (any grade) | Grade 5  adverse event | ≥Grade 3  adverse event | Serious adverse event |
| --- | --- | --- | --- | --- | --- |
| All BPDCN patients | 9 (81.8) | 9 (81.8) | 0 | 2 (18.2) | 4 (36.4) |
| Capillary leak syndrome | 6 (54.5) | 6 (54.5) | 0 | 2 (18.2) | 4 (36.4) |
| Hypoalbuminemia | 6 (54.5) | 6 (54.5) | 0 | 0 | 0 |
| Treatment-naïve BPDCN | 7 (100.0) | 7 (100.0) | 0 | 1 (14.3) | 3 (42.9) |
| Capillary leak syndrome | 4 (57.1) | 4 (57.1) | 0 | 1 (14.3) | 3 (42.9) |
| Hypoalbuminemia | 6 (85.7) | 6 (85.7) | 0 | 0 | 0 |
| Relapsed/refractory BPDCN | 2 (50.0) | 2 (50.0) | 0 | 1 (25.0) | 1 (25.0) |
| Capillary leak syndrome | 2 (50.0) | 2 (50.0) | 0 | 1 (25.0) | 1 (25.0) |

n (%); adverse events were coded using MedDRA ver. 26.1

BPDCN, blastic plasmacytoid dendritic cell neoplasm

**Table S4 Incidence of adverse events related to hypersensitivity (SAF)**

|  | All patients (N=11) | Treatment-naïve  (N=7) | Relapsed /refractory  (N=4) |
| --- | --- | --- | --- |
| Adverse events related to hypersensitivity | 5 (45.5) | 3 (42.9) | 2 (50.0) |
| Rash | 2 (18.2) | 2 (28.6) | 0 |
| Drug eruption | 1 (9.1) | 0 | 1 (25.0) |
| Flushing | 1 (9.1) | 0 | 1 (25.0) |
| Infusion related reaction | 1 (9.1) | 1 (14.3) | 0 |
| Grade ≥3 adverse event related to hypersensitivity | 0 | 0 | 0 |

Adverse events were coded using MedDRA ver. 26.1.

# **Study group**

These were the affiliations of the study group for Tagraxofusp at the time of the study.

Nobuhiro Hiramoto, Department of Hematology, Kobe City Medical Center General Hospital, Kobe, Hyogo, Japan.

Masahiro Onozawa, Department of Hematology, Hokkaido University Hospital, Sapporo, Hokkaido, Japan.

Senji Kasahara, Department of Hematology, Gifu Municipal Hospital, and Laboratory of Pharmaceutical Health Care and Promotion, Gifu Pharmaceutical University, Gifu, Gifu, Japan.

Noboru Asada, Department of Hematology and Oncology, Okayama University Hospital, Okayama, Okayama, Japan.

Toko Saito, Department of Hematology and Cell Therapy, Aichi Cancer Center, Nagoya, Aichi, Japan.

Toshiro Kawakita, Department of Hematology, NHO Kumamoto Medical Center, Kumamoto, Kumamoto, Japan.

Toshio Kitawaki, Department of Hematology, Graduate School of Medicine, Kyoto University, Kyoto, Kyoto, Japan.

Toshihiro Miyamoto, Department of Hematology, Faculty of Medicine, Institute of Medical Pharmaceutical and Health Sciences, Kanazawa University, Kanazawa, Ishikawa, Japan.

Akihiro Hirakawa, Department of Clinical Biostatistics, Institute of Science Tokyo, Bunkyo-ku, Tokyo, Japan.

Yasuhito Terui, Department of Hematology, Saitama Medical University Hospital, Iruma, Saitama, Japan.

Junji Suzumiya, Department of Hematology, Koga Community Hospital, Koga, Shiga, Japan.

Shuichi Miyawaki, Division of Hematology, Tokyo Metropolitan Ohtsuka Hospital, Toshima-ku, Tokyo, Japan.
